# Composted Municipal Green Waste Infused with Biocontrol Agents to Control Plant Parasitic Nematodes—A Review

**DOI:** 10.3390/microorganisms9102130

**Published:** 2021-10-11

**Authors:** Franciska Tóthné Bogdányi, Krisztina Boziné Pullai, Pratik Doshi, Eszter Erdős, Lilla Diána Gilián, Károly Lajos, Paola Leonetti, Péter István Nagy, Vitantonio Pantaleo, Renáta Petrikovszki, Bozena Sera, Anikó Seres, Barbara Simon, Ferenc Tóth

**Affiliations:** 1 ImMuniPot^®^ Independent Research Group, H-2100 Gödöllő, Hungary; 2 Doctoral School of Plant Sciences, Hungarian University of Agriculture and Life Sciences, H-2103 Gödöllő, Hungary; kriszti.pullai@gmail.com (K.B.P.); petrencsi@gmail.com (R.P.); 3 Doctoral School of Biological Sciences, Hungarian University of Agriculture and Life Sciences, H-2103 Gödöllő, Hungary; erdos.esztike@gmail.com (E.E.); karoly.lajos@hotmail.com (K.L.); 4 Szent István Campus Dormitories, Hungarian University of Agriculture and Life Sciences, H-2103 Gödöllő, Hungary; gilian.lilla.diana@uni-mate.hu; 5 Bari Unit, Department of Biology, Agricultural and Food Sciences, Institute for Sustainable Plant Protection of the CNR, 70126 Bari, Italy; paola.leonetti@cnr.it (P.L.); vitantonio.pantaleo@cnr.it (V.P.); 6 Department of Zoology and Ecology, Institute for Wildlife Management and Nature Conservation, Hungarian University of Agriculture and Life Sciences, H-2103 Gödöllő, Hungary; nagy.peter.istvan@uni-mate.hu (P.I.N.); seres.aniko@uni-mate.hu (A.S.); 7 Department of Environmental Ecology and Landscape Management, Faculty of Natural Sciences, Comenius University in Bratislava, Ilkovičova 6, 842 15 Bratislava, Slovakia; bozena.sera@uniba.sk; 8 Department of Soil Science, Institute of Environmental Sciences, Hungarian University of Agriculture and Life Sciences, H-2103 Gödöllő, Hungary; simon.barbara@uni-mate.hu

**Keywords:** sustainability, circular economy, plant parasitic nematodes, *Meloidogyne*, organic soil amendments, municipal green waste compost, biocontrol agents, soil microbiome, soil suppressivity, microbial infusion, RNAi, mycorrhizae

## Abstract

The last few years have witnessed the emergence of alternative measures to control plant parasitic nematodes (PPNs). We briefly reviewed the potential of compost and the direct or indirect roles of soil-dwelling organisms against PPNs. We compiled and assessed the most intensively researched factors of suppressivity. Municipal green waste (MGW) was identified and profiled. We found that compost, with or without beneficial microorganisms as biocontrol agents (BCAs) against PPNs, were shown to have mechanisms for the control of plant parasitic nematodes. Compost supports a diverse microbiome, introduces and enhances populations of antagonistic microorganisms, releases nematicidal compounds, increases the tolerance and resistance of plants, and encourages the establishment of a “soil environment” that is unsuitable for PPNs. Our compilation of recent papers reveals that while the scope of research on compost and BCAs is extensive, the role of MGW-based compost (MGWC) in the control of PPNs has been given less attention. We conclude that the most environmentally friendly and long-term, sustainable form of PPN control is to encourage and enhance the soil microbiome. MGW is a valuable resource material produced in significant amounts worldwide. More studies are suggested on the use of MGWC, because it has a considerable potential to create and maintain soil suppressivity against PPNs. To expand knowledge, future research directions shall include trials investigating MGWC, inoculated with BCAs.

## 1. Introduction

### 1.1. The Importance of PPNs

Plant parasitic nematodes (PPNs), and among them especially, members of endoparasitic genera including *Meloidogyne* (root-knot nematodes, RKNs), *Heterodera* and *Globodera* (cyst nematodes) and *Pratylenchus* (root lesion nematodes) are factors of crop production that should be considered in agriculture and horticulture worldwide. The 10 most important nematode taxa were compiled by Jones (2013) [1]: (1) root-knot nematodes (*Meloidogyne* spp.); (2) cyst nematodes (*Heterodera* and *Globodera* spp.); (3) root lesion nematodes (*Pratylenchus* spp.); (4) the burrowing nematode *Radopholus similis*; (5) the stem and bulb nematode *Ditylenchus dipsaci*; (6) the pine wilt nematode *Bursaphelenchus xylophilus*; (7) the reniform nematode *Rotylenchulus reniformis*; (8) the dagger nematode *Xiphinema index* (the only virus vector nematode to make the list); (9) the false root-knot nematode *Nacobbus aberrans*; and (10) the foliar nematode *Aphelenchoides besseyi*.

The symptoms their infestation may cause are often mistaken for signs of water stress, nutrient deficiencies, soil-related conditions or even bacterial or fungal infections [2].

Attacking their host, PPNs inject enzymes within their saliva, and a feeding site begins to develop within the plant tissue. As a result of the combination of chemical signals and mechanical injuries, physiological changes begin to take place. Cell walls may begin to proliferate, the area of plasma membrane may increase, and these changes make sure that the nutrients taken up by the plant are directly or indirectly transported to the parasite. Galls, cysts or lesions are formed. As a result of biochemical and morphological modifications, water and nutrient uptake and efficacy of use change. Plant development is stunted, productivity drops and the plant begins to yellow and wilt. Additionally, potential and alarming characteristics of PPN infestation are that due to mechanical deformations, the probability of secondary infections by soil-borne pathogen agents is increased; the resistance to these infections is reduced; hence, the potential damage by soil-borne pathogens is increased [3,4,5,6,7].

Since PPNs affect a wide variety of crops of agricultural and horticultural importance, including staple crops, yield losses are estimated to reach as high as 15% of all losses in agricultural production, totaling about USD 125–157 billion [8,9,10].

### 1.2. Conventional Methods to Control PPNs

Pesticides, specifically nematicides, are effective and the most common short-term management options against most PPN species [11,12].

As current European legislation restricts the use of many, formerly reliable pesticides, it is an incentive to find alternative methods and procedures to preserve the environment and the agricultural landscape without endangering the economy [13,14] (Table 1).

### 1.3. New, Alternative Directions

Different organisms including fungi, bacteria viruses, predatory nematodes, insects, mites and some invertebrates have been reported to parasitise or prey on nematodes [48]. The potential of artificially introduced microorganisms or of the soil microbiome to control PPNs is a topic of increasing interest. The extensive use of synthetic fertilisers and pesticides has negative consequences in terms of soil microbial biodiversity and environmental contamination. Faced with this growing concern, a proposed alternative agricultural method is the use of microorganisms as biofertilisers. Many works have been focused on bacteria, but the limited literature on yeasts and their potential ability to safely promote plant growth has gained particular attention in recent years. Some of their positive effects could be due to the provision of soluble nutrients and the production of different phytohormones. Biological control is another mechanism in which antagonistic microorganisms and enzymes that directly contribute to plant growth act as biostimulants [49].

Additionally, different microorganisms such as endophytes, symbionts, pathogens and plant growth promoting rhizobacteria (PGPR) function together with the overall soil microbiome to influence plant health and crop productivity [50]. Although the soil microbial community performs essential roles, there is still a limited understanding about the complexity of the microbial diversity in controlling plant pathogens and pests, as pointed out by Lupatini et al. (2017) [51]. However, recent studies on microbiomes associated with soil, plant roots and PPNs have helped increase understanding of the functional potential of a disease-suppressive microbial cohort by bringing this topic into the limelight [52].

The rhizosphere acts as platform where most of the interactions between different microbial communities and PPNs take place. During the interaction between plants and PPNs, there is a possibility that the microbial community acts as a mediator, which then determines the infectivity of PPNs on plants [52]. As stated by Topalović and Heuer (2019) [53], PPNs encounter different soil microbiota while travelling towards host plants before infection. During this journey, members of the diverse microbial community may attach to them and cause deleterious effects to their cuticle and surface coat. For instance, Bent et al. (2008) [54] investigated soil biological activity against *M. incognita* in two susceptible hosts, a dwarf tomato and wheat in greenhouse trials. Six soils were tested for their biological activity and after combining them, they found a reduction of 5–6-fold in the root-knot nematode population when compared to an identical but pasteurised soil. They identified 11 fungal phylotypes associated with nematodes, although not all of these were correlated with the reduction in nematode population densities.

### 1.4. Objectives

We recognise how the research community explored the background behind using compost, and compost made of MGW in particular, in the management of one of the most important pests of agricultural production, PPNs. We acknowledge the highlights of the scientific results achieved so far and point out why composted materials have an important role in supporting soil suppressivity. We discuss the most recent uses of MGWC against PPNs. Finally, we suggest unmapped research directions.

## 2. The Role of Soil Microbiome

### 2.1. The Importance of Soil Microbiome for a Functional Soil

Soil organisms (the edaphon) create a unique, natural and dynamic ecosystem with distinct properties and the ability to ensure the growth and development of plants [55]. Edaphic conditions are influenced by environmental changes, soil structure and also, through management practices that can alter productivity and soil characteristics including structure and proneness to erosion, disruption and aggregation [56].

Favourable interactions between plants and microorganisms were described as early as in the first half of the 20th century by Katznelson et al. (1948) [57], but regardless of our expansion of knowledge, these interactions still remain hotspots of soil biology [58,59,60].

In a well-functioning soil, soil microorganisms interact with the meso- and macrofauna of the soil and form a functional unit within their environment while shaping this environment as well [61] to ensure a continuous flow of substances and energy through the soil: decomposition and synthetic processes, the processes of transformation of individual elements and nutrients and interactions between soil and its surroundings.

However, the latest, ecological and integrated approach differentiates between the assembly of microorganisms and soil microbiome. The first can be studied focusing on one special taxa or niche, but the term microbiome refers to all its members: alive microorganisms and non-living members, including metabolites or other, functioning molecules. The microbiome functions within a specific set of specific features, including their physical, chemical and biological micro-environment, their specific habitat in which they “interact with each other, live in the same habitat, and form their ecological niche together“, as described by Berg et al. [62].

The soil microbiome is an invisible component of the soil, yet it forms an extremely important functional team, decomposing dead matter and returning nutrients to the circulation, where the released nutrients not only serve plants, but they are also immobilised in the bodies of soil organisms. The presence, activity and interaction of the soil microbiome are important for the proper and full functioning of the soil, also known as “soil health” [63]. The size, diversity and stability of the soil microbiome, together with the stored soil organic matter, are the basis for the stability of the entire soil ecosystem.

### 2.2. The Effect of Spatial Heterogeneity within the Soil on the Abundance of PPNs

When composted MGW enters the soil, the distribution of organic material becomes unavoidably uneven. This uneven distribution of organic material results in an uneven species composition and distribution of PPNs. If we have knowledge on the preferences of various PPN species, we may predict the locations where these pests are most likely to appear in large numbers, so that we may target their control.

The spatial distribution of PPNs of agricultural or horticultural areas is often distinctively uneven: there are “hot spots” and “cold spots” within the same field with the same crop and same management [64,65]. Spatial heterogeneity has long been investigated, and the influence of different factors, mainly different soil properties on the patchy distribution of PPNs, has been the subject matter of several studies.

Brodie (1976) [66] showed that the seasonal vertical distribution of the three PPN species, *Belonolaimus longicaudatus*, *Pratylenchus brachyurus* and *Trichodorus christiei*, varied with soil depth, moisture, temperature and texture. Noe and Barker (1985) [67] showed that 40–50% of the spatial variation in population density levels of the PPN species *Meloidogyne incognita, Tylenchorhynchus claytoni* and *Helicotylenchus dihystera* were related to different soil parameters such as texture, moisture, organic matter, pH or concentration levels of different anions or cations. Similarly, Howland et al. (2014) [68] found that soil moisture had significant effects on the population densities of *Meloidogyne hapla* and *Pratylenchus* spp. within vineyards. The findings of Kandel et al. (2018) [69] in fields of different crops rotations also indicate that soil organic carbon and soil wetness play an important role in determining the spatial distribution of *Pratylenchus* spp. densities. The study of Kawanobe et al. (2020) [70], carried out in sugarcane fields, also showed that the abundances of *Pratylenchus* spp. were significantly influenced by soil pH and K^+^ concentrations and that the abundances of *Tylenchorhynchus* spp. were significantly affected by soil pH and clay content.

Higher pH values, higher N availability and lower P content of the soil was linked to a decreasing occurrence of *M. incognita* in a betel field by Mondal et al. (2018) [71], while the general abundance of PPNs was positively correlated with the organic carbon content of the soil. As organic matter content increased, so did the abundancies of some genera, namely *Paratylenchus, Typenchus* and *Criconemoides* [72].

Results from the study of Avendaño et al. (2004) [73] also indicate a strong correlation between soil texture and the population density of the soybean cyst nematode (*Heterodera glycines* Ichinohe). Similar findings regarding the effect of soil texture were reported by Hbirkou et al. (2011) [74] for beet cyst nematodes (*Heterodera schachtii* Schmidt) within sugar beet fields and by Holguin et al. (2015) [75] for reniform nematodes (*Rotylenchus reniformis* Linford and Oliveira) within cotton fields. The study of Ortiz et al. (2010) [76], which was also carried out in cotton fields, reported that the population density of the southern root-knot nematode (*Meloidogyne incognita* (Kofoid & White) Chitwood) was strongly correlated with various soil properties, especially with the soil electrical conductivity. In another study, the relationship between electrical conductivity and the occurrence of *Meloidogyne* spp. was defined as a weak one (Krif et al., 2020) [72].

A positive correlation between *Pratylenchus neglectus* and electric conductivity was found by Yavuzaslanoglu et al. (2012) [77] while sampling several crops. Their study also documented that different species of the same genus have different preferences towards soil texture. While higher abundancy figures of *Pratylenchus thornei* were only slightly related to clay content, *P. neglectus* had a definite preference towards sandy locations. Quist et al. (2019) [78] also reported that distribution patterns of various genera *Coslenchus*, *Pratylenchus* and *Tylenchorhynchus* may not be the same, regardless of those genera all being polyphagous plant parasites. Sandy soils were more frequently inhabited by members of genera *Helicotylenchus*, *Tylenchorhynchus*, *Longidorus* and *Criconemoides*, whereas *Xiphinema* and *Meloidogyne* spp. were found more often in predominantly clay soils [72].

Besides the abundance or population density, the beta diversity of PPN communities is also affected by different soil properties, especially soil physiochemical variables, followed by soil texture and edaphic type [79]. A multivariate analysis conducted in the study of Quist et al. (2019) [78] showed that soil texture had a stronger impact on soil nematode communities than land management. They also demonstrated that the average geostatistical range (=patch diameter) of 48 nematode taxa in their study fields was related to soil organic matter content. They found that r-strategists (fast-growing bacterivores and fungivores) were positively correlated with soil organic matter content, while most K-strategists (slow-growing omnivores and carnivores) were negatively affected by this parameter.

Crop variety was also found to influence the occurrence of certain PPN genera in a study carried out in a betel vine plantation. Members of the genera *Meloidogyne* favoured the bitter-leaved plants, while *Helicotylenchus* and *Tylenchus* were abundant under the sweet varieties [71]. An investigation into the relationship between certain mineral content within the soil and the distribution of plant parasitic nematodes by Krif et al. (2020) [72] revealed that higher levels of Mg and Mn generally promoted the occurrence of PPNs; some genera, *Helicotylenchus*, *Pratylenchus*, *Ditylenchus*, *Longidorus* and *Criconimoides*, were more influenced by the presence of K and the C/N ratio. The abundances of *Trichodorus* spp., *Rotylenchulus* spp., and *Meloidogyne* spp., on the other hand, were linked to Zn, Cu, P and Na to a greater extent.

The aforementioned physical and chemical soil characteristics influence microbial composition, spatial and temporal diversity [80]. When Signorini et al., (2021) [81] investigated the microbial (fungal and bacterial) diversity using targeted amplicon sequencing, they found that soil heterogeneity resulted in significantly different microbial diversities among samples taken from within vineyard rows, inter-row areas and the headland. As the headlands and rows studied underwent different agricultural practices over the years, their physio-chemical characteristics became different, and so did their bacterial and fungal communities. The hypothesis that there is a definite connection between soil characteristics and bacterial diversity was tested by Curd et al. (2018) [82], who concluded that soil heterogeneity, soil resource availability in particular, is a major contributing factor to alpha and beta-diversity. Earlier still, the effect of spatial heterogeneity of a corn field was found to have a greater effect on the diversity of arbuscular mycorrhizal fungi than any other variable measured [83]. Additionally, Mann et al. (2019) [84] established a connection between management and environmental factors to the abundance and composition of microbial life within the soil while studying soil health parameters. Lower management intensity or the application of manure resulted in higher respiration and wet-stable aggregates, and also in higher indices of mycorrhizal and non-mycorrhizal fungal communities. Heterogeneity within the physical and chemical characteristics of the soil highly correlated with the establishment of location-specific fungal communities, and as the diversity of the mycobiome increased, so did crop yields [85].

Soil heterogeneity, both natural and management-induced, was proven to manifest in the physical, chemical and microbiological dimensions. While there are areas that harbour higher microbiological diversity and activity, other areas of the same field may display a simpler microbiological status, and conventional agriculture may induce losses to biodiversity. Earlier, Culman et al. (2010) [86] noted that the more intense management measures are, the greater the decline in species richness and diversity. However, it is not only diversity that is lost. Following the argument of Briones (2018) [80], we assume that when taxonomical and functional diversities within a spot decrease, predation and competition decrease, too.

### 2.3. Soil Suppressivity

The connection between belowground microbiological diversity and the suppressivity against PPNs was confirmed by Steel and Ferris (2016) [87], who examined nematodes and their natural control agents. They found that under preferable soil conditions, the abundance and biomass of predators increase, and therefore, their suppressivity increases, too. The most frequently cited definition belongs to Baker and Cook (1974) [88], where in a suppressive soil, the pathogen may “establish or persist, but causes little or no damage, but thereafter the disease is less important, although the pathogen may persist in the soil.” According to de Corato (2020) [89] soil suppressivity is the natural ability of soil to suppress soil-borne pathogens and to promote a balanced development root growth. Topalović et al. (2020) [90] describes disease-suppressive soils as the conspicuous prototype of a microbe-mediated plant defence mechanism against the infection of pathogens, including PPNs.

The general suppressivity of a soil stems from the activity of the total microbial biomass in the soil. General suppressivity, therefore, is not transferable between soils, but over time, at the other end of the suppressivity spectrum, more effective specific suppressivity takes over. Owing to the biocontrol activities of microorganisms, specific suppressivity is transferable. With specific suppressivity, one or a few groups of microorganisms may be responsible for biocontrol activity, but interactions with other members of the rhizosphere community can significantly influence the degree of suppressivity [91,92]. On the contrary, general suppressivity is a result of competitive activities of the total micro- and macroflora and is universal to all type of soils. The specific suppressivity of soil is mechanistically complex, requiring the enrichment and activation of a narrow subset of microorganisms and antagonistic traits that inhibit the infection cycle. Interactions between pathogen, host plant, and soil microbiome are also necessary to evoke specific suppression [93]. The overall soil suppressivity measured in organic fields is probably a combined effect of general and specific suppression [94].

### 2.4. Soil Microbiome and Soil Suppressivity

Inhibiting activity towards plant pathogens is the result of biotic or abiotic activities in the soil by several mechanisms. The definition of components associated with suppressive properties against PPNs is sparse when we look at soil as a complete system. Several factors such as physical, chemical and biological factors can induce soil suppressivity. Most research has focused on suppressivity by biological factors, which can also be achieved and proved by applying small amounts of a suppressive soil into a conducive one [95]. More than 100 years of research on suppressive soils has resulted in the accumulation of very fundamental data, but soil microbiome investigations have been strongly promoted since only 2011 [96]. Disease-suppressive soils are exceptional ecosystems owing to the activities of soil microorganisms [97]. Several studies showed soil suppressivity against nematodes or diseases in natural soil samples compared to sterilised and re-infested soil [98,99]. This draws attention to the biological factors and role of the soil microbiome as one of the main components of suppressive soils [96,100].

The microbiome that induces and maintains suppression may be related and attached to the pathogen organism [90,98,101] or to the plant rhizosphere [52,102].

While most previous studies focused on certain microorganism species rather than total soil microbiome [93,98,103,104], it is becoming widely accepted that a complex microbial ensemble can be more effective than a separated strain [105]. Several studies attributed soil suppressivity to indigenous soil microbes [98,106]. Industrially produced microbial strains often fail to establish or survive in the rhizosphere because of the competition with the indigenous soil microbiome; hence, this approach is less effective when applied in large-scale agricultural production [93].

Suppressive soils are resources of microorganisms with novel and beneficial antimicrobial traits used in the area of plant protection. The bacterial profiles of suppressive and conducive soils for certain plant diseases were compared, and as a result of this, suppressive soils showed greater abundance of it [107]. Topalović et al. (2020) [52] observed that lower damage of PPNs on the susceptible plant is related to a positive interaction between the infected plants and their beneficial root microbiota.

With an increasing efficiency, analytical methods have been promoted since the 1990s to clarify which microorganisms or microbial groups are responsible for soil suppressivity. The development and expansion of molecular methods facilitate the detailed characterisation of the biological background of soil suppressivity [108]. Direct extraction methods, without the need of a previous cultivation of the microorganism, enable one to analyse a much greater part of the soil microflora [109]. Molecular and chemical technologies and the application of community and metagenomic analysis methods now may allow one to compare extensively microbial consortia of suppressive and conducive soils beyond the description of a microbial genera [93,110].

## 3. MGW as an Organic Amendment

### 3.1. Introduction to Municipal Green Waste

As the dimension of urbanisation accelerates, so does the amount of waste generated. Municipal solid waste (MSW), which accounted for 46% of the total waste produced in 2009 [111], is an umbrella term, because it stems from various municipal activities including agriculture, urban life and public sectors, with households being one of the important contributors [112]. Globally, there are about 2 billion tons of MSW created every year [113]. The concept of municipal green waste (MGW), on the other hand, is not as homogenous and conclusive. MGW is defined as a “compostable fraction” of MSW or “organic waste”, and the term clearly covers garden waste including leaf litter, grass clippings and pruning waste [111], and according to the level of income of a country, MGW accounts for 28 to 64% of the total waste. Another estimation suggests that MGW can be calculated at a general level of 50% of all municipal waste [114]. However, as the objectives and data sources of papers differ, definitions are different, too, and so the terms “organic fraction” or “organic biodegradable waste” of MSW, although almost identical to the former terms, refer exclusively to food waste of manufacturing, household and catering origin in another paper [113]. For the purposes of this review, we define MGW by its origin and composition.

### 3.2. Generation and General Composition of MGW

Domestic properties, households, by municipal green areas and natural reserves all generate MGW [115,116]. By weight, green waste was found to make up to 22–30% of the total MSW in Taiwan in 2006 [117] and at an average, 66% of MSW in Shiraz City, Iran [118]. In Germany, the yearly generated amount of combined organic waste (garden waste and other, compostable waste) was 103 kg per person [119]. The same data are 151.9 kg for Shiraz City [118] and 120 kg for England [120], which totals to 14.4 million tons per year for the UK [121]. The amount of MGW collected has a considerable variation over countries and within a country, locally, according to the location being urban or rural, and to local gardening practices and waste management traditions [119,120,122].

The content of MGW displays a definite seasonality. Summer collection is dominated by grass cuttings, spent flowers and vegetables, fall by leaves, whereas late fall to spring MGW mostly consists of pruning waste and other woody material [120,122,123].

Not all garden waste produced will be gathered by the municipalities, even where kerbside or other collection methods are available. Firstly, no waste is produced unless it is collected in the garden, as organic materials naturally decompose over time in the exact location they are left [122]. Secondly, once collected by the gardener, organic materials may be burnt or composted on site. This is the case in 56 and 16% (by weight) of generated waste in rural and in urban areas, respectively [120].

### 3.3. Handling and Further Use of MGW

The sustainable and efficient management of MGW should start with the selection, or preferably, with the separate collection of waste materials. Combined with reduced waste generation and recycling [113,114,119,124,125,126], these steps are designed to allow for circles of the circular economy to be closed, as promoted by the New Circular Economy Plan of the European Commission, CEAP in March 2020, where waste is considered as a starting point for converting a material into a valuable resource for further use [127].

Before we look into how MGW is composted, we briefly scan other management options, namely landfill, incineration, recycling and other options, including the anaerobic production of biogas. The percentage ratios of these options have changed over time everywhere, with recycling and composting gaining increasing ratios. In Hungary, for example, less than half as much MW was incinerated, and less than one-eighth of MW ended up in landfills as was composted or recycled in 2019, when compared to figures of the late 1900s [128]. While dumping or landfill without any further use is still in practice, this practice raises environmental, social and financial concerns both in Europe (Wales) and in Southern America (Brazil) [121,129].

Before composting, MGW may be piled up, but here, softer components usually start to decompose, adversely influencing the actual composting process [130,131]. Wherever climatic conditions allow [112], incineration rates of MGW keep increasing. The production of “green electric power” usually involves MGW, which, as a raw material, is greatly valued for its low sulphur content [132,133]. Biogas production, an anaerobic process to transform MGW, is considered an environmentally friendly method that meets the new EC requirements of circular economy [113,114]. In Hungary, the MGW of households is collected kerbside, separately from other household wastes. The amount of incinerated MGW for the purposes of biogas production in Hungary was low at 2% in 2012, but this figure is expected to rise in the future [134].

Composting MGW is a management option where the components that made up MGW return the most closely to their original location: soil. Composting is an aerobic process, where after mechanical shredding, MGW is composted alone or with other materials of organic origin, usually for convenience or to compensate the low moisture content, low N/C ratio or the high lignin and tannin content of bark and other woody materials within MGW. Co-composting means the inclusion of food waste or other, easily degradable material to aid microbiological decomposition processes during composting [117,131,135,136,137,138,139,140].

In Hungary, unfortunately, a significant proportion of compost made of MGW is incinerated in electric power plants (unpublished, confidential information), meaning that the beneficial impact of this valuable material had not yet been depleted to its maximum by the agricultural sector.

In the United Kingdom, on the other hand, where landfill is not a suitable destination, MGW is composted, and among others, is used for nature reclamation projects to support the growth of endemic vegetation [121].

A short investigation into the natural fertilisers in the Hungarian market revealed that there are indeed products that use MGW among their raw materials. The exact volume per volume weight (% *v*/*v*) MGW contents of the products were not always available, but gathering these data was not among the objectives of the review. Besides MGW, these natural fertilisers usually contain other organic materials of municipal or agricultural origin (Table A1).

## 4. The Significance of Compost Made of MGW (MGWC)

### 4.1. Composting MGW

Green fertilisation and the use of green compost are among the traditional, well-established methods that are now being rediscovered. For example, one of the oldest composting plants with controlled technology in Europe has been operating from 1912 in the Czech Republic [141]. Composting is a preferable option to treat organic wastes to obtain a final stable sanitised product that can be used as an organic amendment [142]. One of the possible ways to support plant growth and soil health can be the use of composted MGW in agriculture. This may solve the issue of ever-growing amounts of municipal waste and provide biological support and protection [142,143,144] at the same time.

The decomposition process of MGW is influenced by the physical, chemical and microbiological properties of the original raw materials and soil. These properties include, in particular, the C:N ratio, nutrient content, humidity, temperature, pH value, oxygen content, porosity, grain size and particle size [145]. The properties of compost can be significantly influenced by the structure and species of composted plants. Lignin content, for example, creates physical and chemical barriers to enzymes and microorganisms [139,146]. Decomposition also depends on the arrangement of plant tissues. Monocotyledonous and dicotyledonous plants or herbs and woody plants are differently prone to decomposition, which may be influenced by their anatomical differences.

The two major constituents of MGW are grass clippings and wood waste. Grass is a large volume material and usually has an optimal C:N ratio for composting. Unfortunately, its structure does not allow for thorough aeration, and also, grass clippings do not contain suitable microflora for composting, so for ideal decomposition, it needs to be mixed with different types of raw material, where wood waste is ideal. Lignocellulosic substrate, obtained from wood chips in the maintenance and disposal of urban greenery, from crushed tree bark or from cut straw of cereals or oilseeds, improves the porosity and natural ventilation of the matured compost [139,146,147].

Careful composting techniques are required to achieve and maintain the beneficial effects of MGWC. Co-composting, for example, was found to increase product degradability and nutrient release capacity [148], reducing storage time before shredding, and the initiation of the composting process may save nutrients, especially C-content, from being broken down [149].

### 4.2. Microorganisms in the Composting Process

Soil microorganisms participate in the decomposition of organic matter from the beginning to the final finest phase, i.e., nutrient mineralisation, in which they play an irreplaceable role [147,150]. During decomposition, they colonise the sliced substrate and help to decompose especially hard-to-decompose polymers, such as cellulose, lignin and chitin [151]. Cropped, microorganism-digested and enriched organic matter is more digestible and more attractive to other, soil-dwelling animals. Intensive cooperation between animals and the soil microbiome also takes place in the digestive tract of animals, especially in animals consuming difficult-to-degrade polymers. In the digestive tract, often in certain parts of their intestines, animals have a reservoir of beneficial microorganisms [152]. These microorganisms help digest food, which, together with undigested residues (excrement), enter the environment. A food that is particularly difficult to digest is re-consumed, either by the same individuals or by their offspring. This leads to the more efficient decomposition and use of food, but also to the sharing of beneficial decomposing microorganisms within a community [153].

Major successional changes are generated and influenced by the synergistic interaction between the functional groups of the decay community and the chemical composition of the decaying material [150]. The initial pioneering phase is associated with the development of fast-growing fungal representatives of the Zygomycetes group, using readily available sugars [151]. At this stage, atmospheric nitrogen fixators are also developed, the activity of which improves the nutritional and growth status of the community, which is reflected in the increased biomass of the community, the attraction of zooedaphon and the subsequent development of ammonification bacteria. Zooedaphon participates in the decomposition of litter by its fragmentation and enrichment of the decomposing microbial community with other species [145,152]. The next phase concerns the decomposition of more complex plant components and is associated with the development of cellulolytic microorganisms and nitrifying bacteria. Basidiomycetes are able to use the most degradable substances of the lignocellulosic complex type in the advanced stage of decomposition [151]. The final phase, in which the components of the litter are mineralised, assimilated or transformed into soil organic matter, participates in a typical soil form, especially from the ranks of fibrous fungi and actinobacteria.

### 4.3. The Impact of MGWC on Soil

The benefits of composted or vermicomposted organic waste or farm compost on soil is well documented. Introducing composted organic waste has a notable influence on the physical, chemical and biological parameters of soil, its proneness to erosion, water retention, water availability to plants, soil respiration and bulk density. It improves soil structure and soil aggregation. As higher amounts of nitrogen and carbon are mineralised, the amount of extractable phosphorous increases, too. The addition of compost increases soil organic carbon and total N content, the amount of major (N, P, K) and minor elements (Ca, Mg, Na, K), and nutrient availability [112,142,148,154,155,156,157,158,159,160].

Compared to non-treated, control soil, vermicomposted household waste changed the C/N ratio from 21 to 32, and increased the content of nitrate from 78 to 134 mg/kg, of ammonium from 14 to 139 mg/kg, of phosphorus from 92 to 521 mg/kg, and of potassium from 142 to 1912 mg/kg [161].

These findings suggest that the careful and well-planned addition of MGWC to soil is an important tool to improve the ecological and environmental aspects of agriculture, while keeping the main aim, the production of healthy crops in a desired amount.

### 4.4. The Impact of MGWC on Crops

The addition of MGW or MGWC was found to improve the yield of four crops, including fodder crops and vegetables intended for human consumption, namely fodder beet (*Beta vulgaris* L. ssp. vulgaris var. crassa), forage maize (*Zea mays* L. ssp. mays), Brussels sprouts (*Brassica oleracea* L. var. *gemmifera*) and potato (*Solanum tuberosum* L.), due to the enhanced availability of nutrients, soil organic matter and organic carbon content [154]. Bernal et al. (2017) [156], however, expresses their worry that since nutrients are both present in their organic and inorganic form, their availability is rather uneven, and this might be a problem to any crop. When treated with MGWC, various tree species had a different reaction to the treatment. The development and survival of Common Alder (*Alnus glutinosa* L.) was lower, but Silver Birch (*Betula pendula*, Roth) and European Larch (*Larix decidua* Mill.) throve in the microenvironment presented by MGWC [121]. While vermicomposted organic materials contain compounds that improve crop quality, Sim and Wu (2010) [140] also warn of the dangers of overdosing, as they may cause undesirable side effects. When vermicomposted, the high availability of nutrients is achieved by the biochemical changes nutrients undergo by the gut microorganisms of earthworms [157]. Incorporating MGWC into the soil helped spotted gum tree (*Corymbia maculata*) individuals to overcome water stress and this contributed to the general growth of plants [158]. Sayara et al. (2020) [142] point out how important it is to handle raw materials properly to achieve a stable compost that can raise soil fertility just as well and be a healthy alternative to conventional, chemical fertilisers.

### 4.5. Compost Microbiome

The composting process furthers decomposition, and through mixing, sieving and in many cases, acidification, compost matures and thus, its microbial composition changes. As Pot et al. (2021) [162] observed, among the variables, raw materials had the highest influence on the microbiome of the end product. While the maturation of compost definitely increased microbial diversity, blending and acidification are tools that modify and optimise the compost microbiome.

The fact that composted materials have their own microbial community and that suppressivity is partly attributable to the innate microbiome of compost was suggested by Borrero et al. (2004) [163]. They found that a special combination of alive bacteria, actinomycetes and fungi was detected in *Fusarium*-suppressive composts, and heating or other, biocide manipulations to compost resulted in the reduction in its suppressivity. Compost has an extremely species-rich microbial complex, and the relationship between the resource materials of compost, the maturity of the compost, and the content (predominantly, the amount of readily available nutrients) have been investigated [156].

The pH range of raw MGW and garden waste is between 6.83 and 9.01, whereas composted or vermicomposted MGW and garden waste ranges between pH 7.12 and 7.67 [164], meeting the requirements of bacteria (pH 6.0–7.5), fungi (pH 5.5–8.0) and actinomycetes (pH 5.0–9.0), as described by Gomez-Brandon et al. (2008) [165].

Postma et al. (2003) [144] studied the densities of microorganisms of garden and organic household waste, both constituents of MGW, and found that there were aerobic bacteria, bacterial spores and filamentous actinomycetes in both fresh and mature composts, regardless of the origin, whereas fluorescent pseudomonads, fungi and *Trichoderma* species were characteristic to the garden waste, regardless of its age.

Several antagonist fungi and bacteria have been observed in compost, including *Trichoderma, Penicillium, Aspergillus, Bacillus, Pseudomonas*, and *Actinomycetes* species [166], which play a role in control of several soil-borne pathogens and PPNs [167,168,169]. The major taxa responsible for soil suppression are mainly nematophagous bacteria, including *Bacillus, Pseudomonas, Stenotrophomonas*, and *Streptomyces* [170,171]. Of nematophagous fungi, one of the most studied taxa is *Trichoderma* spp. [172,173,174]. The establishment and survival of these BCAs are directly and indirectly influenced by abiotic factors such as soil texture, moisture, nutrients, organic matter and pH [175]. Another study observed the occurrence and colonisation of nematophagous fungi in compost [176], including *Arthrobotrys oligospora*, capable of changing to a predatory lifestyle and producing structures that trap and kill nematodes [177,178,179]. In addition, other fungi, including *Verticillium chlamydosporium* and *Trichoderma* spp., are able to parasitise nematode eggs [167,180,181].

Nematode antagonist fungi can include nematophagous fungi and endophytic fungi and are capable of infecting and killing different developmental stages of nematodes, thus reducing their population density [182].

Nematophagous bacteria are also ubiquitous and have a wide host population [183], so they have been used in the control of nematodes [184,185]. During their life processes, they produce antibiotics and toxins that inhibit nematode reproduction and survival, or directly cause nematode death via the lysis of their cell walls [186]. Endophytic bacteria colonise internal plant tissues without causing symptoms and protect plants from pathogens [187,188]. *Pseudomonas* sp., *Bacillus thuringiensis*, and *Streptomyces avermitilis* are among the most studied nematophagous bacteria [189].

Several *Paenibacillus ehimensis* strains have been shown to be antagonists of plant parasitic nematodes [190] because they produce a hydrolytic enzyme [191]. Nematophagous bacteria also produce other, non-toxic enzymes, including chitinases, proteases and gelatinase, which may kill nematodes or damage eggshells [192]. Several bacterial strains also produce volatile organic substances that induce systemic resistance to PPNs in plants [193].

Numerous studies have shown that compost improves soil quality due to increased organic matter content and soil microbial populations, and it has also been shown that it improves plant resistance to plant parasitic nematodes [188,194,195]. In addition, several studies have reported a decrease in the number of PPNs after composting [196]. Compost contains microbes that are antagonistic to nematodes, and compost combined with plant-derived nematicidal compounds can have a significant effect on the development of the PPN population [197].

### 4.6. The Impact of MGWC on Microbial Life

More than a decade ago, there was already solid interest in the effect of the addition of composted materials and soil amendments on PPNs, and the potential of compost in the control of PPNs. At the same time, however, the effect on the soil microbiome and microbial communities received less attention [198].

Although microbial activity plays a crucial role in the formation and maturation of compost [146], and the addition of organic amendments increases microbial biomass [154,160,199] and may induce major changes in the species composition and abundance of microbial communities [200], partly due to its strong correlation with its high organic matter, Ca, Mg and K content [155], the biological interactions between the soil microbiome and the microbial species composition of organic amendments, including MGW, have not yet been satisfactorily explained. The reason for this may be, for example, the different content (quality and quantity) of biologically active substances in MGW, which depends on the plant-species composition of MGW. Plant bodies contain various amounts of cellulose, chitin, flavonoids, lignin, phenols, ricin, saponins, tannin, terpenoids, etc., which significantly affect the growth of microorganisms and thus affect the resulting cooperating soil microbiome during the decomposition. The addition of compost was shown to increase the overall nematode presence, while modifying species composition, favouring bacterivorous taxa and decreasing the ratio of fungivorous nematodes [201].

Kurzemann et al. (2020) [202] observed that compost type influenced the changes to the composition of the soil microbiome, and the impact was hardly detectable on fungal species, while more pronounced on bacterial assemblages. A compost made of a mixture of rice bran and crab shells had a significant effect on the bacterial community of the rhizosphere [203]. A similar experience was recorded when green waste compost was added to soil in a biosolarisation study, as the impact of compost was definitely more evident and comprehensive for bacteria, and the changes to fungi were less noticeable [204]. The impact of compost on soil bacteria was present and uniform, regardless of the type of compost applied, when the soil treated with compost made of various raw materials, including urban organic waste, green waste manure or sewage sludge [160]. On the other hand, when only the fungal community was investigated, some fungal taxa were supported, while others were weakened by the addition of compost, as expressed in terms of their relative abundance. The effect of compost was noticeable in different depths and within-field locations of soil as well [205]. Vermicomposted household waste modified the ratios of fungi and bacteria (e.g., fungi/bacteria from 0.18818 to 0.00425). Compared to control soils, a significant, 2-to-4-fold increase took place in the number of beneficial nematodes belonging to bacterivorous, fungivorous and predatory groups, with no change in the abundance of plant-parasitic nematodes [161].

These findings suggest that the addition of compost has a selective effect on the soil microbiome, and this “selectivity” or “preference” may not only be present at a taxonomic but also at a functional level. In a long-term, follow-up study, for example, compost-amended grasslands were found to have less bacteria and archaea that are important for nitrification processes within soil. On the other hand, taxa that either have a role in carbon exchange or promote plant growth increased in abundance [199]. Achmon et al. (2020) [204] also reported a highly significant drop in the relative abundance of plant pathogenic fungal taxa. These results, in turn, may imply that compost is able to “re-write” the microbiome of a soil, or replenish a partially missing or dysfunctional microbiome, and thus, the addition of compost can be used to achieve specific goals, as suggested by Azeem et al. (2020) [200] in the case of turfgrass soil.

### 4.7. The Background Mechanisms by Which MGWC May Enhance Microbial Life

By what mechanisms can compost and composted MGW enhance microbial life? As Tognetti et al. (2008) [148] noted, as the degradability of carbon and the total nutrient content increased, these stimulated the activity of microorganisms, which is shown by the increase in *q*CO_2_, the metabolic quotient, the rate of soil microbial respiration per unit of microbial biomass increases [160], although changes to *q*CO2 were not always consistent with the addition of compost [155], but equally as important is the increase in water soluble carbon, which is an easily available nutrient source. As soil organic carbon content rises, the process of mineralisation and immobilisation of nutrients progresses; D’Hose (2014) [154] points out that these changes, in turn, will improve the functionality of the soil, as improvement to soil characteristics concurred with an increase in microbial biomass responsible for decomposing organic matter and nutrient cycling, more specifically, the number of earthworms, the relative abundance of bacterivorous nematodes and a definite drop in both in the total number of individuals and the relative abundance of PPNs. The addition of composted materials induces significant changes to the physical and chemical properties within the soil [199], and when a diverse spectrum of organic matter is found in abundance within the soil, microbial density rises [92]. At the same time, however, no connection was detected between compost application and the measures of predatory, fungivorous and omnivorous nematode taxa [154]. Considering environmental conditions, bacterial and fungal communities benefit from the elevated levels of soil moisture due to MGWC, and therefore can reach higher microbial abundance [158].

## 5. MGWC, a Tool against PPNs

### 5.1. The Concept of Using Composted Materials against Pests and Pathogens

It has long been known that many plants exude compounds that are either repellent to nematodes or have a direct nematode-suppressive effect. These plants have been sown or used as green manures to protect the targeted, susceptible crop, and this effect can be achieved with the addition of compost or vermicompost made of beneficial plants, including Brassicaceae as well as Asteraceae species [206]. Ikwunagu et al. (2019) [207] found that biochar made of organic materials of plant and animal origin reduced the viability of eggs of the root-knot nematode (*Meloidogyne* spp.).

In addition, secondary metabolites contained in plant bodies may also play a role. A positive effect on the quality of compost in terms of reduction in undesirable soil diseases and pathogens [196] has been recorded. For example, the composting of plants of the Brassicaceae family affect against root-knot nematodes [208]. The secondary metabolites of the Brassicaceae family, glucosinolates, coexist in vivo with glycosylated thioglucosidases, which are responsible for their hydrolysis and isothiocyanate production. Glucosinolates and isothiocyanate act as defensive bioactive metabolites against plant pathogens, insects and herbivores. Compost from plants of this family has a positive effect on the number of the most common PPNs in the soil [208]. Botanicals as a combination of organic compounds was found to be effective against *M. incognita* and reduced the number of J2 larvae and the severity of galling in tomato [209].

A study on the suppressive capabilities of formulated plant biomasses or waste materials to the root-knot nematode *M. incognita* biocontrol [210] demonstrated that soil treatments with formulated plant biomasses or waste materials can be an effective alternative to green manure crops for the sustainable management of PPNs infestations.

The addition of organic materials was found to improve soil conditions and create a healthier matrix for functional microbial communities, and reduce the impact caused by a wide range of pests, including bacteria, soil-borne pathogens, and PPNs [143,196,211]. Achmon et al. (2020) [204] conclude that the presence of soil organic matter can contribute to the suppression of soil-borne pathogens.

Biostimulants are natural substances or microorganisms that are applied to plants with the aim of improving their nutritional efficiency, tolerance to abiotic stress and/or quality traits of crops, regardless of their nutrient content. Biofertilisers, a subcategory of biostimulants, can be defined as microbial inoculants, in which active or inactive formulations of beneficial microorganisms can improve the nutritional efficiency of plants [212,213,214]. The addition of PGPR increased resistance to nematodes in a specially formulated potting medium for muskmelons [215], but as suggested by a recent finding, plant-growth-promoting agents may only enhance the growth and increase the biomass of crops when the experimental area, a greenhouse, was abundantly supplied with nutrients [216].

Research has shown that compost contains a variety of microbes that can control PPNs in different ways during their life processes [171,172,173,217].

Additionally, when soil suppressivity was recovered by the addition of a *Trichoderma* strain, crops benefited from the presence of the fungal agent when the availability of water and nutrients was increased by the addition of composted green waste [218]. In yet another study, *Rhizoctonia solani*, a bacterial disease, was successfully suppressed by the addition of compost and a fungal antagonist [219]. These observations suggest that compost may not only be used along with biological control agents, but compost may also carry BCAs and encourage their action.

With a suitable carrier, the application of BCAs will be easier, but the most important motivation to find a carrier is the striking difference between BCA survival and virulence in a controlled environment and in open fields. The reliability of BCAs drops among realistic conditions because BCAs are highly sensitive to biotic and abiotic factors, because not only extremities, but fluctuations of environmental conditions can be harmful [220]. Yet, the most frequently suggested solution is not the search for a carrier medium, but the selection of more resistant strains, genetic engineering, formulation, encapsulation or packaging techniques, combination with organic pesticides and the introduction of synthetic microbial communities [52,221,222,223,224,225].

Among BCAs, antagonists of arthropods pests and plant pathogens were given the majority of attention, while antagonists of PPNs were somewhat neglected. In one of the most recent publications on fungal biocontrol agents, Joos et al. (2020) [226] observed that *T. harzianum* was able to survive and keep its virulence after having spent a certain time period in compost.

In contrast, while the idea to use compost as a matrix to aid the survival of entomopathogenic nematodes (EPNs) was first voiced in 1999 [166], compost as a carrier of EPNs is still a fresh topic of investigation. Outlining the drawbacks of applying the EPNs the conventional way, and testing green waste compost obtained from local municipalities, Herren et al. (2018) [227] found that a well-aged, biologically mature compost provides a sheltering medium for EPNs from the risks of adverse environmental conditions and enhances their survival and virulence, making their application in agricultural production more effective.

### 5.2. RNA Interference (RNAi)-Based Technology and BCAs

One of the most sophisticated methods of controlling PPNs depends on RNA silencing, an RNA-mediated mechanism in eukaryotes that regulates gene expression on the transcriptional or post-transcriptional level and decisively affects multiple biological processes [228]. Gene silencing through the increased degradation of mRNA represents a cellular pathway that is functional in a broad range of organisms. The discovery of this important regulatory process comes from the experimental introduction of RNA into cells to interfere with the function of an endogenous gene [229]. Only a few molecules of injected double-stranded RNA were required to trigger the interference with gene expression, suggesting that there could be a catalytic or amplification component in the interference process.

In the last two decades the role of RNA silencing in host-antiviral defence and transposon silencing has been established, suggesting a potential application in functional genomics of plant breeding, since RNA silencing is a major immune response of plants against several pathogen taxa, including viruses [230,231]. Viral infection generates viral-derived double-stranded (ds)RNAs, which, in turn, induce antiviral RNA silencing. Dicer-like RNaseIII (DCL) proteins detect and process viral dsRNAs, and thus, duplexes of small interfering viral RNAs (vsiRNAs) of various sizes, i.e., 21, 22 and 24 nucleotides in size, are generated. When infection occurs, vsiRNAs accumulate and act as silencing signals. This may be amplified by the activity of plant RNA-dependent RNA polymerases (RDRs) [232]. The RDR-generated primary and secondary vsiRNAs may induce a local and systemic immunity, and this in turn will reduce the level of viruses [233]. While the RNA-induced silencing complexes (RISCs) are incompletely described [234], the most important active components are the Argonaute nucleases (AGOs). When a vsiRNA is incorporated into an AGO protein, the guide strand remains and leads the RISC to the cognate viral RNA. Through slicing, AGO1 and AGO2, two of the best-known effectors, contribute to the removal of viral RNA and/or subviral entities. The endonucleolytic cleavage of the target RNA is sliced in a sequence-specific manner, governed by vsiRNA [234,235]. A similar defence system can be triggered against PPNs, as described below.

More recently, several works have shown that endogenous siRNAs from coding genes and non-coding genes are involved in RNA-silencing-based antiviral immunity, resulting in a widespread gene downregulation and plant resistance to pathogens [236,237]. Specific RISCs may also mediate silencing of DNA viruses via DNA methylation [238]. RNA silencing also plays a pivotal role in plant defence against pathogenic fungi [239].

In studying the impact of biocontrol on PPNs, we find it particularly important to highlight the best and most efficient gene targets for silencing genes related to the interaction. To achieve this, functional genomics methodologies, including genome sequencing and transcriptome profiling, are employed [240].

Many PPN species have been used in RNA gene silencing studies [241], and the success of RNAi in cyst- and root-knot nematodes has been demonstrated, and more than twenty successful applications were reviewed and summarised by M. N. Rosso et al. in 2009 [242].

RNAi technology was used for the stable production of siRNAs in plants to downregulate the MiDaf16-like1 and MiSkn1-like1 genes of *M. incognita*, which are orthologous to DAF-16 and SKN-1 transcription factors (TFs) in *Caenorhabditis elegans* during host plant parasitism. For the development of the biotechnological nematode control of *M. incognita* by the means of the number of eggs, galls, J2 and the reproduction factor (NRF), two TFs were proven to be potential targets [243].

One of the most promising and targeted approaches to the biocontrol of parasitic nematodes in crops is RNA interference [244]. In describing the strategy against the cereal cyst nematode *Heterodera avenae* by using RNAi-inducing streptomycete-derived biostimulants in *Triticum aestivum* L. plants, the authors demonstrate the synthesis of si/miRNAs within wheat cells and their silencing activity by dot blot hybridisation experiments, concluding that these biostimulants are able to reduce nematode infestation through plant-induced nematode mortality. The most effective and direct way should be to select specific genes in the PPN that are silenced by si/miRNAs produced in host plants under the action of biostimulants [244,245]. This is to underscore what is increasingly emerging from the recent literature, namely that plant miRNAs can be exploited to inhibit pathogen development or aid mutualistic relationships. Just as the host immune response can be suppressed by transferring miRNAs from eukaryotic organisms to plants. This two-way exchange could be the future in exploiting miRNA-based technologies for agricultural practices [246].

In conclusion, RNAi is critical for regulating host immunity against bacteria, fungi, oomycetes, viruses and PPNs. Similarly, sRNAs from pathogens and pests also play an important role in modulating their virulence and knowledge in cross-kingdom RNA trafficking, and environmental RNAi may be developed into novel effective strategies to fight diseases caused by microbial pathogens and pests [247].

### 5.3. Compost and MGWC as an Environment for PPNs

The management of PPNs by means of soil organic amendments, including composts, has been studied for a long time. Despite the multitude of literature that refers to this topic, exact comparison is difficult, due to the high variation in the applied substances and terminologies. Therefore, in the following section, only some of those resources are reviewed where composted municipal green waste materials were certainly involved.

As a part of an extended series of examinations, Renčo et al. (2010) [248] studied the effects of five different compost mixtures on soil nematode community structure in a pot test using a natural grassland soil sown with barley (*Hordeum vulgare* L.) seeds. The duration of the experiment was 5 months. Out of the five compost treatments, two were relevant to this review: ‘C_3_’ constituted a mixture of urban green residues and sewage sludge (70:30) and ‘C_4_’ was mixed from urban green residues and soil (90:10), respectively. The ratio of composts added to experimental soil was 0, 1, 1.5, 5 and 10 percent. Regarding the two treatments involving municipal waste, significant negative effects were found in all nematode feeding groups, except predators whose density did not decrease as compared to the control plots. These treatments appeared particularly suppressive to bacterial feeders and omnivores. Moreover, PPNs were the most heavily suppressed by ‘C_4_’, i.e., the mixture of MGW and soil, while ‘C_3_’, i.e., MGW and sewage sludge, showed a gradually increasing negative effect along the higher application rates. In particular, genera *Bitylenchus* and *Helicotylenchus* decreased along the increasing rates of ‘C_4_’ treatment, while *Merlinius* responded with an increasing density. As for ‘C_3_’ treatment, the overall decrease along the application rates was due to the amounts of *Rotylenchulus* whose numbers in the plots with the maximum application rate shrank to below half of that in the lowest treatment, reaching figures equal to those in the control plots. All the other genera showed some increase along the gradient of the application rates, with *Bitylenchus*, *Helicotylenchus* and *Merlinius* remaining below their levels in the control plots, while *Paratylenchus* and *Pratylenchus* exceeded those slightly or to a remarkable extent, respectively. These findings on the deleterious effects of MGW compost on PPNs were confirmed by a further study by Renčo et al. (2009) [249], where the densities of genera *Bitylenchus, Helicotylenchus, Heterodera, Paratylenchus* and *Rotylenchulus* were found to decrease along a gradient of increasing application rates of MGW compost.

On the other hand, green waste composts are highly variable, and the presence or absence of certain substances can fundamentally alter the effects of a compost in controlling PPNs. The addition of leaves or fruit pericarp of the common walnut (*Juglans regia*) to compost, for example, is an issue that generates constant debate, as these green materials contain juglones that the literature considers harmful to soil biota. An early summary by Akhtar and Mahmood (1994) [250] lists 120 different plants species used in PPN control, but interestingly, walnut is not included. Nevertheless, a working group in India repeatedly found low concentrations (2% and below) of juglone extracts to be highly toxic to *M. javanica* [251] and *Meloidogyne incognita* Race 2 [252]. Similarly, Jakusovszky et al. (2019) [253] found the extracts of freshly fallen walnut leaves to be lethal to *Meloidogyne incognita*, with a mortality value of 95% in a concentration of 0.78% and total mortality in all the higher concentrations tested. On the other hand, the same concentration of 0.78% left the slug-parasitic nematode species *Phasmarhabditis hermaphrodita* unaffected, and only the higher concentrations from 1.56% onwards caused a mortality of 80–100% on this species. Furthermore, on the bacterial feeder nematode species *Panagrellus redivivus*, even a concentration of 6.25% failed to cause a significant increase in mortality as compared to the control.

Apart from PPNs, both the formation and application of compost deeply affect other nematode feeding groups as well, particularly bacterial and fungal feeders. Moreover, the composition of these groups may offer a promising tool to indicate compost maturity. Therefore, Steel et al. (2018) [254] proposed a Nematode-based Index of Compost Maturity based on four nematologically relevant criteria: (i). nematode abundance, (ii). a ratio of fungal and bacterial feeding nematodes, (iii). the presence of more than one fungal-feeding taxon and (iv). the presence of diplogasterid nematodes, a particularly important, predominantly bacterial feeding group. After the necessary tests and refinements, NICM may be widely used a useful method to assess compost maturity.

In the light of the above detailed findings, it is important to survey the effects of composted MGW on PPNs while keeping in mind the complexity of the influenced system. For example, antagonistic effects from other nematode feeding groups, also influenced by the treatments, should be considered as well. In a comprehensive literature study, Thoden et al. (2011) [255] found several organic soil amendments, including composts, to pose ambivalent effects on PPNs. Besides the often-observed decline, populations of PPNs sometimes remained unaffected or even increased in certain cases, but it does not necessarily affect crop yields in a negative way. As the authors concluded, the overall positive effects of organic amendments on crop yields can be attributed to various factors apart from the toxicity of organic amendments to PPNs, including the enhanced plant growth stimulated both directly and indirectly by various antagonists of PPN populations.

### 5.4. The Role of Arbuscular Mycorrhiza Fungi (AMF) and Composted Materials in the Control of PPNs

The role of arbuscular mycorrhiza fungi (AMF) in the water and nutrient uptake of cultivated plants is well documented, especially when water and nutrient availability is poor [256,257]. The mutualistic relationship between AMFs and plants is governed by the plants [258]. The presence of AMF on the roots was shown to enhance the resistance of plants against PPNs [259], or other root herbivores [260] and even affected pollinators through the changes to floral traits [261]. There are various regulatory mechanisms in the background. On one hand, AMF and certain nematodes compete for the colonisation of cortical cells, and also, AMF act as a physical barrier. On the other hand, AMF-inoculated plants have an improved nutrient status, and therefore, can be more resistant [259]. Furthermore, AMF can induce a systemic resistance in plants against nematodes [262]. In a field experiment, PPNs and AMF showed negative co-occurrence in the root rhizosphere [263] as a result of the competition for this specific habitat. Benedetti et al. (2021) [264] found that the presence of AMF (*Glomus etunicatum*) decreased the number of female cyst nematodes (*Heterodera glycines* Ichinohe) with 28.21% in the roots of soybean in a greenhouse experiment. Additionally, AMF-inoculated plants had 26% greater shoot yield when compared to plants without AMF. The authors suggest that the AMF was able to improve the nutrient status of soybean, and that led to a higher tolerance of PPNs. Compost also has a positive effect on the growing and nutrient state of plants, so probably, a simultaneous application of AMF and compost can induce an ever-higher PPN-resistance in plants. Amerany et al. found (2020) [265] a synergetic effect among chitosan, compost and AMF. Although AMF and low doses of compost applied together inhibited mycorrhizal colonisation, the root, shoot biomass, root length and the leaf area of tomatoes were greater than the same parameters in control plants. The reason for this phenomenon could be the better availability of phosphorus in the presence of the medium-dose of compost, which led to the deactivation of the AMF system. The same results were found in a glasshouse experiment, where the higher rates of green waste-derived compost decreased AMF colonisation [266], while AMF improved the Zn and P supply of tomatoes, especially by low rates of compost. Other experiences with the combined application of compost and AMF may have contradictory results. When Jan et al. (2014) [267] examined the effects of AMF and compost from animal dung and rock phosphate on berseem (*Trifolium alexandrinum*) yield and P uptake, the maximum root colonisation of AMF was measured by the full dose of compost and the maximum spore number was found by the half dose. The increased yield and better nutrient uptake underline the role of AMF inoculation and compost in berseem. There is little available information about the interrelationship among AMF, PPNs and compost. Galal et al. (2012) [268] conducted a greenhouse experiment to examine the combined effect of different biocontrol agents (*Streptomyces antibioticus, Bacillus subtilis,* AMF, *T. harzianum* and *P. penetrans*, compost) against the root-knot nematode (*M. javanica*), and concluded that due to synergism, combinations are more effective than single usages of any of the BCAs. Unfortunately, the combination of AMF and compost alone was not included in this study. Rizvi et al. (2018) [269] demonstrated that the simultaneous usage of *Glomus fasciculatum* and bio-organic waste (*Avena sativa* straw) resulted in larger plants and a lower size of nematode population (*M. incognita*) in chickpea (*Cicer arietinum* L.), and the effect can be attributed to the involvement of other microorganisms (*Mesorhizobium ciceri, T. harzianum).*

Based on these results, we suggest a further, more detailed examination of the multimicrobial inoculation of compost as the most promising, eco-friendly alternative of PPN control.

### 5.5. Earthworms May Have an Indirect Control on PPNs

Earthworms play a crucial role in mixing organic amendments with the mineral phase of the soil, thus deepening the topsoil and improving soil health. When vermicomposted, organic materials decompose faster due to earthworm activity. Faster decomposition and the presence of earthworms influence microbial activity. Reviewing the literature on the interaction between earthworms and root herbivores, Wurst (2010) [270] concluded that earthworms can compensate for the negative effects caused by root-feeding nematodes on plants. Treatments with earthworm influence (vermicompost) can decrease root-feeding nematode abundance and plant damage caused by above-ground herbivores. Xiao et al. (2016) [271] studied the effect of conventional compost and vermicompost using the earthworm *Eisenia fetida* on root-knot nematodes (*M. incognita*) in a climate chamber with a susceptible and a resistant tomato cultivar. Although more root galls were found on susceptible plants compared to resistant ones, vermicompost was more efficient in the protection against RKNs compared to conventional compost, especially in the case of susceptible plants, while root galls on both susceptible and resistant tomato plants significantly declined under conventional compost and vermicompost treatments compared to inorganic fertiliser at 14- and 30-days post inoculation. The liquid phase extract of vermicompost, vermiwash, contains several compounds, decomposing bacteria, fungi, vitamins, enzymes, mucus and skin secretion produced by earthworms, and as the earlier literature confirms, it can aid the suppression of diseases and decrease the growth of pathogenic bacteria and pests. Therefore, vermiwash, either incorporated into the soil or sprayed on the plants, can effectively help protect crops from bacterial and fungal pathogens and pests [272].

Regarding the method of application of plant residues, Tao et al. (2009) [273] studied the effects of earthworms on nematode communities with plant residues incorporated into the soil or placed on the top as mulching. When they compared the earthworm-worked treatments (mulched or incorporated) with treatments without earthworms, they found significantly lower total nematode abundances in all the examined soil depths after residue incorporation in earthworm-worked treatments. When the residue was only placed on the top of the soil, lower total nematode abundance was found in the topsoil layer compared to the treatment without earthworms; however, the difference was not significant.

Demetrio et al. (2019) [274] reviewed studies assessing the interaction between earthworms and soil nematodes in a meta-analysis. They found that there was a 27% reduction in soil nematode density due to earthworm activity, with PPNs reduced by 24%, free-living nematodes by 26%, and there was no significant effect on fungal-feeding nematode densities. Epigeic and anecic earthworm species had the greatest reduction effect on nematodes, while soil dwelling endogeic and epi-endogeic forms did not have significant effect. All the examined earthworm densities significantly decreased nematode populations; however, greater densities caused larger decreases. Regarding management type, earthworms had a negative effect on all nematode forms (average 27% decrease) in a greenhouse, while in field experiments, they only had a negative effect on the free-living nematodes (16%).

### 5.6. The Use and Efficacy of MGWC with or without BCAs against PPNs

#### 5.6.1. The Impact of Composted Materials on Soil Suppressivity

The impact of composted materials on soil, crop yield and soil microbiome has been well documented. With environmental and sustainability concerns on the rise, organic approaches in agriculture have gained more attention. One of the most valued aspects of compost is its ability to mitigate crop loss by controlling soil-borne pests and pathogens [156]. The addition of compost promotes plant health and their nutrient status, establishes and enhances microbial life, and not only the volume of microbial mass, but microbial activity as well [142,148,197,275]. Additionally, the increase in microbial biomass generates competition, leading to suppression [91]. The suppressive ability of composted materials, including yard waste, brewery compost, vineyard waste, cattle manure, wood bark, and other organic materials, has been reported since in the early 1980s, against fungal pathogens including *Rhizoctonia, Fusarium* or *Pythium* [163,276,277,278,279], and soil-borne, immobile pests including PPNs [275,280]. The increase in biomass includes the increase in the antagonists of nematodes, predators and fungal and bacterial parasites [197].

#### 5.6.2. Compost-Induced Suppressivity against PPNs

The addition of composted materials and MGWC may suppress PPNs by enhancing their natural antagonists, as shown by the elevated levels of enzymatic activity of microorganisms [275]. The establishment and build-up of a reliable nematode suppression by an assemblage of antagonistic biological control agents, however, requires a longer time, as suggested by McSorley and Gallagher (1995) [281]. Oka (2010) [197] contemplates that it may not be a collection of antagonistic microorganisms, but compost-derived compounds that contribute to the suppression of nematodes, instead. These compounds may induce resistance in crops or may directly be nematicidal. Compost not only introduces nutrients, but it also increases their availability, and this results in the enhancement of the functional diversity of the soil microbial community [155]. McSorley (2011) [282] also argues that in many cases, the introduction of large amounts of decomposing plant material is a simulation of fertilisation, where crops benefit from increased soil fertility and nutrient availability, and this alone may surpass the effects that the compounds may have on PPNs.

Additionally, since the addition of any composted material improves soil structure, namely particle size, Oka (2010) [197] argues that this change alone is an important factor in soil suppressiveness, simply by allowing natural enemies of PPNs to penetrate and inhabit the newly formed larger pores within the soil.

#### 5.6.3. The Role of Microorganisms in PPN Control

Among microorganisms, beneficial fungi and bacteria may be used in the biological control of PPNs.

A rapidly growing interest in fungi as a biocontrol agent has been observed and their exploitation has gained attention [283]. These fungal biocontrol agents fall under four categories: (1) nematode-trapping or predatory fungi that produce trapping devices; (2) endoparasitic fungi that use conidia to enter the nematode body; (3) parasites that use the host as food source; and (4) toxin-producing fungi that secrete toxins [284]. Below, we review some of the most detailed studied biocontrol agents against PPNs.

The literature on microorganisms with a definite nematode suppressive effect continues to expand. Some of these taxa strengthen plant defence or induce resistance. Documented cases of this effect were reported by Westphal and Becker (2001) [285], and the beneficial agents against *Heterodera schachtii* were, among others, *Fusarium oxysporum*, *Dactylella oviparasitica* and *Paecilomyces lilacinus*. Inoculation with *Glomus intraradices*, *Glomus mosseae* and *Glomus etunicatum* suppressed *M. javanica* in peach [286]. Oka (2010) [197] recounts taxa including Rhizobacteria, namely *Bacillus pumilis* and *B. mycoides, B. sphaericus, Agrobacterium radiobacter, Rhizobium etli*, or fungi including *T. harzianum*, a non-toxic *Fusarium oxysporum* and *Neotyphodium coenophialum*. Zakaria et al. (2013) [287] reported that the fungus *Verticillium chlamydosporium* and the symbiotic bacterium *Photorhabdus luminescens* reduced the preproduction performance and gall formation of *Meloidogyne incognita* in cucumber. The list expanded with *Pasteuria, Pseudomonas* and *Rhizobium* species [102], *Penicillium* sp. and *Aspergillus* sp. [288], the highly investigated *Metarhizium anisopliae* [289] and *Aphanocladium album* [290]. In their compilation, Topalović et al. (2020) [52] refer to *Pasteuria*, *Bacillus*, *Pseudomonas*, *Rhizobium*, *Streptomyces*, *Arthrobacter* and *Variovorax*, or fungal isolates of *Pochonia*, *Dactylella*, *Nematophthora*, *Purpureocillium*, *Trichoderma*, *Hirsutella*, *Arthrobotrys* and *Mortierella* as being successful against PPNs. Kumar and Dara (2021) [291] acknowledge studies over the last few decades and describe bacterial and fungal endophytes, including *Enterobacter intermedius, Lactobacillus paracasei, Chaetomium globosum* and *Bacillus megaterium*, to name a few, that were all found to be antagonistic towards PPNs.

*Trichoderma* spp. is one of the most successful fungal biocontrol agents against PPNs. For instance, Spiegel and Chet (1998) [292] evaluated the efficacy of *Trichoderma* spp. against PPNs and soil-borne fungi. They tested different *Trichoderma harzianum* and *Trichoderma lignorum* isolates against *M. incognita* (Treub, 1885) Chitwood, 1949 in short- and long-term experiments. In the short term, exposure to *T. harzianum* in a nematode-infested soil improved the growth of nematode-infected plants and decreased the root-galling index, whereas the long-term experiment improved the growth and yield of infected plants, but had no effect on the galling index. *Trichoderma asperellum* exhibiting biocontrol activity against *Meloidogyne javanica* was also observed by Spiegel et al. (2005) [293], with direct fungal parasitism as the possible underlying mechanism. The inoculation of tomato seeds with *Trichoderma harzianum* significantly reduced the infestation by *M. javanica* under greenhouse conditions [294], where the fungal agent adversely influenced the establishment, development and reproduction characteristics of the nematode. Khan et al. (2018) [295] investigated both the suspension culture and exudates of *Trichoderma* spp. against *Meloidogyne incognita*. They found that the fungal metabolites had a direct effect on *M. incognita* by decreasing the egg hatching rate and increasing the mortality rate of J2 larvae. The salicylic acid-signalling pathway and ethylene biosynthesis were induced in tomato treated with *Trichoderma harzianum* when infected by root-knot nematodes and limited the infection via the activation of systemic acquired resistance (SAR) and ethylene production [296]. Meanwhile, the soil application of fungal spores had a more diverse impact: not only did they negatively affect the J2 population, but they promoted plant growth more effectively than the exudates.

The nematophagous fungus *Paecilomyces lilacinus* (Thom) Samson is also a promising fungal biocontrol agent candidate against PPNs. Khan et al. (2006) [297] tested *P. lilacinus* and nematode trapping fungus *Monacrosporium lysipagum* (Drechsler) Subram individually and in combination against three economically important PPN species, namely the root-knot nematode *M. javanica*, cereal cyst nematode *Heterodera avenae* Wollenweber, and burrowing nematode *R. similis* (Cobb) Thorne on tomato, barley and banana plants in pot trials. They found that the combined application of these fungi was the most effective treatment and, in some cases, *M. lysipagum* was better than *P. lilacinus* when applied alone. Kiewnick and Sikora (2006) [298] tested *Paecilomyces lilacinus* strain 251 (PL_251_) against root-knot nematode *M. incognita* in growth chambers. They found that treated pre-planted soil reduced the occurrence of root galling by 66%, egg masses by 74% and nematode population by 71%. Additionally, a single pre-planting application of the agent was found to have a successful control of nematodes. The *P. lilacinus* strain 251 was also tested against the northern root-knot nematode *Meloidogyne hapla* Chitwood [299], and the strain PL_251_ reached 90% under favourable temperature conditions, and repeated treatments significantly increased tomato yield, too.

Parasitic or opportunistic parasitic bacteria, rhizobacteria, Cry protein-forming bacteria, endophytic bacteria and symbiotic bacteria are natural enemies of PPNs [192]. Biofertilisers in the form of bacterial nitrogen fixer, phosphate and potassium solubilising bacteria and microbial strains of certain bacteria have been shown to reduce the population of *M. incognita* in chili and tomato, and *Tylenchulus semipenetrans* on Washington navel orange [300]. Topalovic et al. (2019) [301] isolated bacteria belonging to genera *Microbacterium, Sphingopyxis, Brevundimonas, Acinetobacter* and *Micrococcus* that attached to and parasitised *M. hapla*. They concluded that most of the attached bacteria significantly reduced the J2 population on tomato roots. Having said that, one of the most outstanding examples of bacterial control of PPNs is *Pasteuria* spp. [302].

The use of plant growth-promoting rhizobacteria (PGPR) has also emerged as a potential, sustainable solution to control PPNs with the added advantage of producing various plant-growth-promoting substances [303]. Abuzar and Haseeb (2010) [304] investigated two rhizobacteria MTCC and Pf-5 and urea treatments against PPNs in *Cajanus cajan* and found that they significantly reduced the number of PPNs with an increase in plant growth. Improved plant growth, plant defence and biomass of nematode-infested PGPR-inoculated tomato plants along with significant reductions in the J2 population and root galls were observed by Khanna et al. (2019a, 2019b) [305,306] when they tested *Pseudomonas aeruginosa* (M1) and *Burkholderia gladioli* (M2) in tomato against *M. incognita*.

The four most important commercially available biological agents are two bacteria: *Bacillus firmus* strain N1 (a Gram-negative, spore-forming bacteria) and *Pasteuria usgae* (a Gram positive, mycelial endospore-forming bacteria); and two fungi, *Paecilomyces lilacinus* isolate P 251 and *Myrothecium verrucaria*. Additionally, while four *Pasteuria* species have been found to be effective, including *Pasteuria thornei* against root lesion nematodes (*Pratylenchus* spp.), *Pasteuria nishizawae* against cyst-forming nematodes (*Heterodera* spp. and *Globodera* spp.), *Pasteuria usgae* against sting nematodes *(Belonolaimus* spp.), and *Pasteuria penetrans* against root-knot nematodes (*Meloidogyne* spp.), respectively, only *P. usgae* is used in a commercial product [307].

#### 5.6.4. Soil Suppressivity against PPNs Is Initiated and Supported by MGWC

Since the higher availability of nutrients within the soil indirectly encourage saprophytic activities, which, in turn will influence suppressivity [92,308], the addition of composted materials is expected to increase or at least, maintain suppressivity. However, what success was found with the application of MGWC?

Renčo and Kováčik (2015) [309] tested the nematicidal potential of vermicompost and a derivate, vermicompost tea, produced from MGW (30% leaves and 70% grass clippings) with or without urea on potato cyst nematodes *Globodera rostochiensis* and *G. pallida*. The number of eggs and J2 nematodes per cyst were significantly lower on treated plots, and there was a definite difference between the sensitivity of the two species, with *G. pallida* being less sensitive. A compost mix made from crab shell powder, rice bran with the addition of a complex fertiliser, and inoculated with a *Paenibacillus ehimensis* strain was found to reduce the impact of *Meloidogyne incognita* in tomato [202]. Composted MGW incorporated into the soil at various percentages induced an indirect suppressivity within the potting media of barley plants. Unfortunately, this treatment reduced the number of bacterivore and fungivore nematodes as well, leaving only predatory taxa uninfluenced [248].

#### 5.6.5. The Efficacy of Compost-Induced Suppressivity

Apart from a few exceptional cases, given field conditions, the controlling accuracy and reliability of organic amendments are not considered equivalent with the effect of conventional, chemical treatments or fumigants [105,197,224,310].

Although the control effect of organic amendments may be unreliable, it is acknowledged that where such an application is effective, the beneficial consequences last longer than those of conventional treatment options, because the changes to the composition and the activity of microorganisms generate a new environment, which is not as appropriate for a new outbreak of PPN populations [197].

There is a consensus that besides the microbial composition and the actual effect of composted materials, suppressivity partly depends on the raw materials and the preparation (composting) method, various composting additives, the application, farming practices including crop rotation, intercropping, land use, climatic factors and soil properties including the amount, composition and activity of the microbial community, soil type, particle size, C/N ratio, temperature, moisture and oxygen content [144,282,310,311,312,313]. Therefore, future studies should include field experiments to discover ways to guarantee an acceptable efficacy of composts inoculated with beneficial microorganisms among the stressful natural conditions [226].

#### 5.6.6. Establishing and Maintaining Suppressivity with or without BCAs

Harnessing, establishing and maintaining a highly functioning soil microbiome has a great potential for agriculture [62]. Yet, it is a definite challenge to rely on the natural, microorganism-driven suppressivity of an agricultural soil ecosystem. It requires long-term commitment, including a diverse and repeated addition of decomposing organic materials (either by composted MGW or by other means), a reduced soil disturbance to protect the fabric of microbial diversity and the habitat of the agents themselves, and a combination of management techniques to provide the co-existence of predators and targeted pests, as implied by Steel and Ferris (2016) [87]. Suppressivity seems to be a combination of organic supplements and the beneficial microorganisms. Compost contributes to improved soil and plant conditions [144], and when microbial biomass and diversity increases, not all microorganisms act directly against pests and pathogens. Some of them may alter plant response and the presence and actions of harmful organisms, thus contributing to the overall suppressivity, as suggested by Oka (2010) [197].

The most important motivation to find a carrier for BCAs is that while most BCAs display promising performance in a controlled environment, their reliability drops in open fields. There is a troublesome difference between BCA survival and virulence in controlled and realistic conditions, partly due to the higher complexity of the biotic and abiotic factors of the field environment, and to the high sensitivity of BCAs to these factors and their interactions [52].

#### 5.6.7. The Importance of Microbial Diversity in Suppressivity

The diversity and the structure of soil microbial communities are of primary interest when studying soil suppressivity [109]. While physical factors, including soil type, moisture and temperature can influence nematode abundance, soil suppressivity may be also based on nematode population regulation by density-dependent antagonists [99]. The greater the complexity of the biological community of the soil, the greater is the stability of the soil and, consequently, the level of natural biological control [314].

Hussain et al. (2018) [102] found that the diversity of bacterial communities definitely decreased as the investigated area was shifted from bulk soil (with the highest diversity) to rhizosphere and to root endosphere, and finally, to the cyst of *Heterodera glycines*, where bacterial diversity was the lowest. Similarly, Zhou et al. (2019) [315], who tested the impact of soil microbiome on the damage done by PPNs, established that healthy soils showed higher diversity figures then infested soils, and that these two habitats had habitat-specific microbial communities, too.

Can elevated diversity lead to suppression? As Berg et al. (2017) [316] points out, pathogens are integral parts of the biotic environment, and therefore, the onset of soil-borne diseases is made harder when the soil microbial diversity is higher. This agrees with the conclusion drawn by Topalović et al. (2020) [90] that low levels of PPN infection are found simultaneously with higher diversity figures of bacterial and fungal communities. Similarly, where the diversity and abundance of these antagonistic microbial taxa is high, the control of PPNs can be more successful, provided the environmental conditions also favour the establishment of these beneficial microorganisms [315]. In their study, suppressivity was transferred when plants within a formerly conducive soil displayed less severe symptoms after the soil was inoculated with healthy roots and the associated microbiome. There have been attempts at the re-location or re-establishment of suppressivity; conducive soils being turned to suppressive with the help of soil microorganisms was mentioned by Westphal and Becker (2001) [285] as early as in 2001. An interesting reverse example was recorded when the use of antagonistic fungi as bionematicide resulted in increased rhizosphere microbial populations [290].

In their compilation, Bertola et al. (2021) [159] emphasise the importance of higher levels of microbial diversity in curbing the severity of pest occurrence and damage to crops, and they draw attention to the idea of “ecological intensification”. This concept is based on the idea that a naturally enriched soil microbiome provides a more efficient complexity of soil ecosystem services. Comparing an organically managed system to conventional farming system, an increased taxonomic and phylogenetic richness, diversity and heterogeneity of the soil microbiota is observed [51]. Indirectly, this may lead to increased suppressivity, as suppressivity to plant pathogens is mainly related to biotic soil characteristics, represented by the total microbial activity of the soil (FDA hydrolysis) at the pasture, fallow ground and forested areas [314].

However, what species composition and abundance figures are realistic to a compost in action? The compilation by Bernal et al. (2017) [156] clearly states that there is a definite change in the bacterial community composition during composting: certain taxa exist predominantly during the initial phases, others are present throughout the process, and others were reported mainly in the matured compost. Even more interestingly, as maturation progressed, bacterial diversity was found to be either lower or higher at the end of the progress. Similarly, based on earlier findings, Borrero et al. (2004) [163] discusses the importance of diversity as a factor, and comes to the indecisive conclusion that microbial densities, abundance and the total number of microorganisms may not be as important in determining whether a soil performs suppressivity of, or conductivity for, a disease. Additionally, referring to earlier studies, Bertola et al. (2021) [159] resolves the importance of microbial diversity as ambiguous in contributing to a more functional and supportive soil. Topalović et al. (2020) [90] adds to the debate that suppressivity requires the inclusion of a specific set of microbial community.

## 6. Conclusions

**Figure 1:**
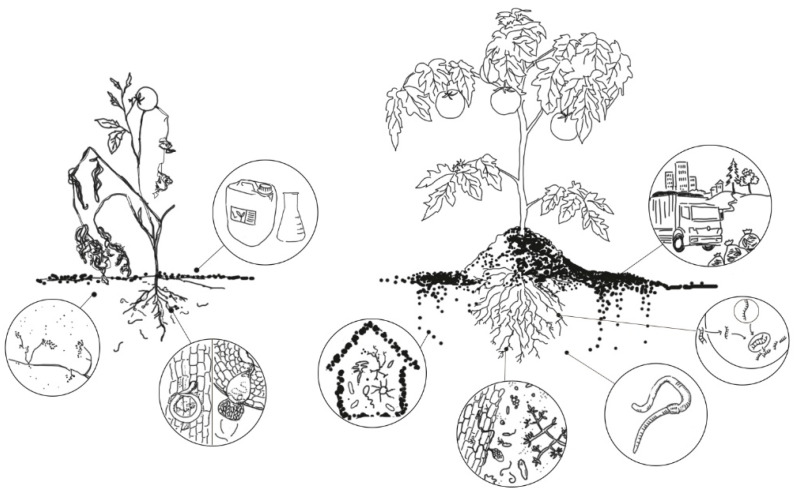
The disadvantageous consequences of conventional, large-scale farming systems and where the natural soil microbiome is taken care of with the use of composted organic waste at farm level. On the left side, the topsoil receives chemical fertilisers and a single-strain microorganism, but no support to enhance or encourage the natural microbiome of the soil. On the right, where composted municipal green waste is applied, with the addition of a consortium of biocontrol agents, the compost serves as substrate to protect the agent; helps the microbial life of the rhizosphere; enhances the defence mechanisms of the crop, attracts more beneficials, including earthworms; provides a field for the RNA interference technology; and gives an alternative for using high amounts of under-appreciated MGW.

There is a striking difference between the status of soil microbiome, the diversity of microorganisms, and overall crop status of conventional, large-scale farming systems and of a farm where composted organic waste, enhanced with beneficial control agents, is repeatedly applied to the soil (Figure 1).

When selecting control methods against PPNs, efficacy is of key importance, but compromising sustainability, ecological safety, and the special cultural and mechanical characteristics of an area [17] may be too high a price for a method that may not work in the long term.

Based on our own experience, the use of mulch and composting materials creates an inappropriate environment for PPNs and helps beneficial nematodes and microorganisms, while also promoting plant growth.

Composting not only reduces the amount of municipal green waste but is an effective and natural way to contribute to environmentally friendly soil remediation. A healthy soil with the right amount and composition of soil microbiome can provide an array of environmental functions, one of which is plant protection. Applying composted green waste is a preventive measure: MGWC has a great potential to create and maintain soil suppressivity against PPNs.

Definitions and approaches of soil suppressivity developed from general observations towards realising the various role of soil microbiome. While the biological factors of suppressivity is a relatively active research area, most studies still focus on selected microorganisms and distinguish them from the total microbial community, but the diversity of microorganisms is fundamental for suppressivity. To understand, conserve or rebuild suppressivity, a holistic approach and farming system using a high amount of organic matter is needed.

Recent compilations [142,313] reveal that the benefits of applying composted organic waste to aid suppressivity against soil-borne pathogens and diseases has been thoroughly investigated and demonstrated. We found, however, that less attention was given to exploring the potential of composted materials against PPNs.

Additionally, the literature on compost as a carrier of BCAs, detailing the background mechanisms by which compost can support the survival and action of beneficial organisms, the conditions of composting procedure, the preparation and inoculation of BCAs and finally, their application, is rather incomplete.

Both the high diversity of applied substances along with their often-unpredictable composition and the variations in naming these materials make it rather difficult to create a general picture on the effects of organic soil amendments applied against plant parasitic nematodes. Yet, some trends are promising and clear: the addition of composted MGW decreased the number of different genera of PPNs, along with other feeding types, sometimes. Therefore, the application of these treatments is supported for nematode control purposes. However, in order to increase both efficiency and environmental safety related to the application of MGW, we advise the involvement of a sort of ‘ecotoxicological aspect’, i.e., studies focused on the nematological effects of common components of composts to learn more about their nematicidal impacts and to possibly avoid side effects on beneficial organisms within the soil.

An emerging new technology, RNAi, can be used to develop deliverables for foliar sprays, soil or seed treatments aiming to control pests and pathogens. The use of MGW infused or not infused with BCA will take advantage of RNA biology studies to define a high level of selectivity, which leads to a reduced risk for non-target organisms and especially respect for the environment to which we are so strongly interconnected.

Through different mechanisms, AMF was found to support crops against PPNs, and the use of compost with the addition of BCAs can enhance this effect. Based on these results, we can suggest a further detailed examination of multimicrobial inoculation with compost as the best eco-friendly alternative of protection against PPNs.

The collection and composting of MGW requires a large labour force, both human and mechanic, and even when composting takes place, composted MGW is a heavy bulk material. Its transportation is extremely expensive, and spreading it evenly on an arable field or even within a protected environment (greenhouse, etc.) is difficult. Not all farmers are open to the use of composted green waste. Education is lacking regarding to what extent weeds or diseased plant materials are rendered harmless via composting. Farmers in general fear the potential harmful consequences of using compost. Compost is a highly variable material, hard to standardise, and therefore policymakers fear the uneven quality of compost, and nor are they familiar with the advantages of composting and the use of compost.

Further field studies involving vermicomposted MGW are needed to explore the efficacy and practical use of the compost product against nematodes.

We conclude that in order to rely on, to re-establish and support the natural defence mechanisms of agricultural soils against PPNs, we have to encourage and enhance the soil microbiome. From the ecological, environmental and microbiological point of view, the use of composted MGW, a valuable resource material produced continuously everywhere, is a long-term and sustainable solution. More studies, including field investigations, are suggested for a more successful application of MGWC, inoculated with BCAs.

## Figures and Tables

**Table 1:** The most important conventional methods to control plant parasitic nematodes.

Method	Feature	Advantages	Main Concerns	References
Chemical methods				
Nematicides	Contact or systemic chemical compounds.	Fast and efficient; high return on high-yielding crops.	Not profitable for low-value crops; needs repeated usage and trained staff; toxic; enters the human food chain.	[11,12,15,16,17,18,19]
Fumigants	Granular or liquid formulations.	More effective than non-fumigant nematicides; long time inactivation.	Phytotoxic; needs coverage.	[15,20]
Non-chemical methods				
Heat	Soil treatment with steam, hot water or solarisation or plant treatment with hot water.	Inactivates nematodes, reduces the number of other pests, diseases and weeds.	High costs; special equipment; harmful to beneficials; releases phytotoxic compounds; possibility of heat damage.	[21,22,23,24,25,26]
Soil cover	The even soil surface is irrigated to soil capacity and covered with a plastic foil.	Efficient to a soil depth of 20 cm; supports crop development and increases yield.	Climatic conditions have a huge impact on efficiency; high costs; contamination by used foils.	[21,27,28,29,30,31]
Plant residues	Organic material is spread on the surface or incorporated into the soil.	Reduces erosion; provides nutrients; recycling.	Uneven efficiency of various plant materials; high costs of spreading high-bulk materials; additional work.	[31,32,33,34,35,36,37]
Crop rotation	Using non-host plants in the sequence of plants.	Effective.	Many host plants; farmers specialise in only a small number of crops.	[38,39,40]
Resistant or tolerant varieties	A result of crop selection and breeding.	Effective for a limited time.	Promotes the emergence of more aggressive and tolerant PPNs.	[41]
Fallow or improved fallow	The field is left empty for 1–2 years, or only leguminous crops are planted.	Reduces the number of PPNs.	Needs weed management; the area becomes prone to water and wind soil erosion.	[16,42,43,44]
Trap plant	Sensitive plants attract PPNs, and these plants are removed before the nematode lifecycle ends.	Inhibits larval development.	Needs labour, time and money.	[45,46,47]

## Data Availability

Not applicable.

## Abbreviations

AMF—arbuscular mycorrhizal fungi; RKN—root-knot nematode; PPN—plant parasitic nematode; MGW—municipal green waste; MGWC—composted MGW; BCA—biological control agent; PGPR—plant growth promoting rhizobacteria; EPN—entomopathogenic nematodes.

## Appendix A

**Table A1:** Compost-based products registered in Hungary, their MGW and total green waste content by volume, when available, and the most important other ingredients. BioMass products are inoculated with BioMass Kappa, which, depending on the type of use, is a solid-phase preparation containing 3–5 × 10^7^–10^9^ useful germs per gram, or a liquid-phase preparation containing 4 × 10^11^ germs per gram. The inoculation constitutes for 5% *v*/*v* of the end product. The FINO FARM HUMUS Extra Humuszkomposzt is inoculated with smaller amounts of compost made within the factory earlier up to 0.5% *v*/*v*. No other products contain inoculants.

Product Name	MGW (% *v*/*v*)	Total Green Waste (% *v*/*v*)	Major Other Ingredients
Agrohum	n/a		MSS, manure, straw
Agromass Kombi	n/a		MSS, straw
Alisca	100		
Berki	100		
BioMass Mikrokomplex Komló	n/a		Ashes from trees, MSS
BioMass Super ASA Organic	27		Organic waste of agricultural origin
BioMass Super Biofuna	n/a		MSS, sawdust
BioMass Super Bonycom	n/a		MSS, sawdust
BioMass Super Csabai	n/a		MSS, straw, hay
BioMass Super ÉRV	n/a	50	MSS
BioMass Super FCsM	n/a		MSS, straw, hay
BioMass Super Kedvenc	n/a	65	MSS, straw, hay
BioMass Super Komszol	n/a		MSS, straw, hay
Biomass Super Ökovíz	n/a		MSS, straw, hay
BioMass Super Sopvíz	n/a	65	MSS, straw, wood shavings
BioMass Super Tápió	n/a	60	MSS, straw, wood shavings
Biomass Super Vital A	n/a		MSS, paper sludge
BioMass Super Vital B	n/a	35	MSS, CPW, straw
CITY	100		
Compostal	100		
CSVK Makói	100		
Depónia	100		
FINO FARM HUMUS Extra Humusz	20	49.5	
Gyöngykomposzt	n/a	80	MSS
Gyulai zöld	n/a		MSS
Haraszti	31.7	40	MSS, woodchips, sawdust
Herculia	n/a		MSS, paper sludge, straw
Hírös Zöld	100		
Kerka	n/a		MSS, straw, wood shavings
Keszthelyi Zöld	n/a		organic waste
Kompvital	n/a		manure, grape marc
Kiskunhalasi	100	66	
MiKomp	100		
Nyírségi Bioföld	n/a		Vegetable and fruit waste
Orosházi zöld	100		Garden shavings
Pannónia	100		
Pécsi zöld	100		
Pro	n/a		Grape marc, wine lees, wood shavings
ProfiKomposzt	100		
Pusztazámori	100		
R	100		
Recomp	n/a		MSS, peat
Regionális szennyvíziszap	n/a	90	MSS
Remusz	1	15	MSS, beer sludge, straw
Speciál	100		
STKH	100		
Szegedi	100		
Szelektív	100		
Szikszói	n/a		
Tisza	100		
Vertikál Adonyi	100		
Vertikál Bácskai	24	25	MSS, straw, wood shavings
Vertikál Csongrádi	30	35	
Vertikál Kalocsai	100		
Vertikál Körös térségi	30	35	
Vertikál Polgárdi	100		
Vertikál Sárbogárdi	100		
Zala	100		Sawdust
Zöld Híd	100		
Zölderő	100		

Abbreviations: MGW: municipal green waste, MSS: municipal sewage sludge, CPW: cellulose production waste, *v*/*v*: volume weight.

## References

[1] Jones J.T., Haegeman A., Danchin E.G., Gaur H.S., Helder J., Jones M.G., Kikuchi T., Manzanilla-López R., Palomares-Rius J.E., Wesemael W.M. (2013). Top 10 plant-parasitic nematodes in molecular plant pathology. Mol. Plant Pathol..

[2] Mesa-Valle C.M., Garrido-Cardenas J.A., Cebrian-Carmona J., Talavera M., Manzano-Agugliaro F. (2020). Global research on plant nematodes. Agronomy.

[3] Rodiuc N., Vieira P., Banora M.Y., Engler J.D. (2014). On the track of transfer cell formation by specialized plant-parasitic nematodes. Front. Plant Sci..

[4] Neher D.A., Powers T.O., Hillel D. (2005). Nematodes. Encyclopedia of Soils in the Environment.

[5] Hernandez Nopsa J.F., Thomas-Sharma S., Garrett K.A., Van Alfen N.K. (2014). Climate change and plant disease. Encyclopedia of Agriculture and Food Systems.

[6] Chindo P.S., Khan F.A., Erinle I.D. (1991). Reaction of three tomato cultivars to vascular diseases in presence of the root- knot nematode *Meloidogyne incognita* race 1. Crop Prot..

[7] Marley P.S., Hillocks R.J. (1996). Effect of root-knot nematodes *(Meloidogyne* spp.) on Fusarium wilt in pigeonpea (*Cajanus cajan*). Field Crops Res..

[8] Watkins P.R., Huesing J.E., Margam V., Murdock L.L., Higgins T.J.V., Altman A., Hasegawa P.M. (2012). Insects, nematodes, and other pests. Plant Biotechnology and Agriculture—Prospects for the 21st Century.

[9] Singh S.K., Hodda M., Ash G.J. (2013). Plant-parasitic nematodes of potential phytosanitary importance, their main hosts and reported yield losses. EPPO Bull..

[10] Singh S., Singh B., Singh A.P. (2015). Nematodes: A threat to sustainability of agriculture. Procedia Environ. Sci..

[11] Hajihassani A., Davis R.F., Timper P. (2019). Evaluation of selected nonfumigant nematicides on increasing inoculation densities of *Meloidogyne incognita* on cucumber. Plant Dis..

[12] Medina-Canales M.G., Terroba-Escalante P., Manzanilla-López R.H., Tovar-Soto A. (2019). Assessment of three strategies for the management of *Meloidogyne arenaria* on carrot in Mexico using *Pochonia chlamydosporia* var. *mexicana* under greenhouse conditions. Biocontrol Sci. Technol..

[13] Hillocks R.J. (2012). Farming with fewer pesticides: EU pesticide review and resulting challenges for UK agriculture. Crop Prot..

[14] Handford C.E., Elliott C.T., Campbell K. (2015). A review of the global pesticide legislation and the scale of challenge in reaching the global harmonization of food safety standards. Integr. Environ. Assess. Manag..

[15] Haydock P.P.J., Woods S.R., Grove I.G., Hare M.C., Perry R.N., Moens M. (2006). Chemical control of nematodes. Plant Nematology.

[16] Adediran J.A., Adegbite A.A., Akinlosotu T.A., Agbaje G.O., Taiwo L.B., Owolade O.F., Oluwatosin G.A. (2005). Evaluation of fallow and cover crops for nematode suppression in three agroecologies of south western Nigeria. Afr. J. Biotechnol..

[17] Phani V.R., Khan M.R., Dutta T.K. (2021). Plant-parasitic nematodes as a potential threat to protected agriculture: Current status and management options. Crop Prot..

[18] Giannakou I.O., Panopoulou S. (2019). The use of fluensulfone for the control of root-knot nematodes in greenhouse cultivated crops: Efficacy and phytotoxicity effects. Cogent Food Agric..

[19] Hussain M., Zouhar M., Ryšánek P. (2017). Comparison between biological and chemical management of root knot nematode, *Meloidogyne hapla*. Pak. J. Zool..

[20] Sikora R.A., Bridge J., Starr J.L., Luc M., Sikora R.A., Bridge J. (2005). Management practices: An overview of integrated nematode management technologies. Plant Parasitic Nematodes in Subtropical and Tropical Agriculture.

[21] Lopes E.A., Dallemole-Giaretta R., dos Santos Neves W., Parreira D.F., Ferreira P.A., Ansari R.A., Mahmood I. (2019). Eco-friendly approaches to the management of plant-parasitic nematodes. Plant Health under Biotic Stress. Volume 1: Organic Strategies.

[22] Roux-Michollet D., Czarnes S., Adam B., Berry D., Commeaux C., Guillaumaud N., Le Roux X., Clays-Josserand A. (2018). Effects of steam disinfestation on community structure, abundance and activity of heterotrophic, denitrifying and nitrifying bacteria in an organic farming soil. Soil Biol. Biochem..

[23] Ferraz S., Freitas L.G., Lopes E.A., Dias-Arieira C.R. (2010). Manejo Sustentável de Fitonematoides.

[24] Marbán-Mendoza N., Manzanilla-López R.H., Manzanilla-López R.H., Marbán-Mendoza N. (2012). Chemical and non-chemical tactics to control plant-parasitic nematodes. Practical Plant Nematology.

[25] Tihohod D. (1993). Nematologia Agrícola Aplicada.

[26] Knoetze R. (2020). The effect of hot water treatment of rooted grapevine nursery stock on the survival of the root-knot nematode, *Meloidogyne javanica* (Nematoda: Heteroderidae). S. Afr. J. Enol. Vitic..

[27] Katan J., Gamliel A., Glinski J., Horabik J., Lipiec J. (2011). Soilborne diseases, control by physical methods. Encyclopedia of Agrophysics.

[28] Liu E.K., He W.Q., Yan C.R. (2014). ‘White revolution’ to ‘white pollution’—Agricultural plastic film mulch in China. Environ. Res. Lett..

[29] Ijoyah M.O., Koutatouka M. (2009). Effect of soil solarization using plastic mulch in controlling root-knot nematode (*Meloidogyne* spp.) infestation and yield of lettuce at Anse Boileau, Seychelles. Afr. J. Biotechnol..

[30] Hou X.-Y., Wang F.-X., Han J.-J., Kang S.-Z., Feng S.-Y. (2010). Duration of plastic mulch for potato growth under drip irrigation in an arid region of Northwest China. Agric. For. Meteorol..

[31] Aminu-Taiwo B.R., Idowu A.A., Alamu O.O., Olaniyi O.W., Olufunmi O.O. (2014). Influence of mulch materials on population of plant parasitic nematode, growth and yield of okra (*Abelmoschus esculentus* L. Moench). IOSR J. Agric. Vet. Sci..

[32] Rahman L., Whitelaw-Weckert M.A., Orchard B. (2011). Consecutive applications of brassica green manures and seed meal enhances suppression of *Meloidogyne javanica* and increases yield of *Vitis vinifera* cv Semillon. Appl. Soil Ecol..

[33] Stirling G.R., Wilson E.J., Stirling A.M., Pankhurst C.E., Moody P.W., Bell M.J., Halpin N. (2005). Amendments of sugarcane trash induce suppressiveness to plant-parasitic nematodes in a sugarcane soil. Australas. Plant Pathol..

[34] Ramezani H. (2013). Management of root-knot nematode, *Meloidogyne incognita* with some organic amendments. Plant Prot. J..

[35] Forge T., Walters T., Koch C. (2014). Use of composted dairy manure solids mulch for raspberry: Influences on soil nematode communities and N and P availability. Compost Sci. Util..

[36] Stavi I. (2020). On-site use of plant litter and yard waste as mulch in gardening and landscaping systems. Sustainability.

[37] Renčo M. (2013). Organic amendments of soil as useful tools of plant parasitic nematodes control. Helminthologia.

[38] Dury J., Schaller N., Garcia F., Reynaud A., Bergez J.E. (2012). Models to support cropping plan and crop rotation decisions. A review. Agron. Sustain. Dev..

[39] Abad P., Favery B., Rosso M.N., Castagnone-Sereno P. (2003). Root-knot nematode parasitism and host response: Molecular basis of a sophisticated interaction. Mol. Plant Pathol..

[40] Fuller V.L., Lilley C.J., Urwin P.E. (2008). Nematode resistance. New Phytol..

[41] Tzortzakakis E.A., Vieira dos Santos M.-C., Conceição I. (2016). An update on the occurrence of resistance-breaking populations of root-knot nematodes (*Meloidogyne* spp.) on resistant tomato in Greece with six new records from Crete. Hell. Plant Prot. J..

[42] Ntidi K.N., Fourie H., Mc Donald A.H., De Waele D., Mienie C.M.S. (2012). Plant-parasitic nematodes associated with weeds in subsistence agriculture in South Africa. Nematology.

[43] Thomas S.H., Schroeder J., Murray L.W. (2005). The role of weeds in nematode management. Weed Sci..

[44] Nielsen D.C., Calderón F.J., Hatfield J.L., Sauer T.J. (2011). Fallow effects on soil. Soil Management: Building a Stable Base for Agriculture.

[45] Westerdahl B.B. (2020). Evaluation of trap cropping for management of root-knot nematodes on annual crops. Acta Hortic..

[46] Cuadra R., Cruz X., Fajardo J.L. (2000). Cultivos de ciclo corto Como plantas trampas Para el control del nematodo agallador. Nematropica.

[47] Navarrete M., Djian-Caporalino C., Mateille T., Palloix A., Sage-Palloix A.-M., Lefèvre A., Fazari A., Marteu N., Tavoillot J., Dufils A. (2016). A resistant pepper used as a trap cover crop in vegetable production strongly decreases root-knot nematode infestation in soil. Agron. Sustain. Dev..

[48] Stirling G.R. (1991). Biological Control of Plant Parasitic Nematodes. Progress, Problems and Prospects.

[49] Hernández-Fernández M., Cordero-Bueso G., Ruiz-Muñoz M., Cantoral J.M. (2021). Culturable yeasts as biofertilizers and biopesticides for a sustainable agriculture: A comprehensive review. Plants.

[50] Chaparro J.M., Sheflin A.M., Manter D.K., Vivanco J.M. (2012). Manipulating the soil microbiome to increase soil health and plant fertility. Biol. Fertil. Soils.

[51] Lupatini M., Korthals G.W., de Hollander M., Janssens T.K.S., Kuramae E.E. (2017). Soil microbiome is more heterogeneous in organic than in conventional farming system. Front. Microbiol..

[52] Topalović O., Hussain M., Heuer H. (2020). Plants and associated soil microbiota cooperatively suppress plant-parasitic nematodes. Front. Microbiol..

[53] Topalović O., Heuer H. (2019). Plant-nematode interactions assisted bymicrobes in the rhizosphere. Curr. Issues Mol. Biol..

[54] Bent E., Loffredo A., McKenry M.V., Becker J.O., Borneman J. (2008). Detection and investigation of soil biological activity against *Meloidogyne incognita*. J. Nematol..

[55] Wolters V. (1991). Soil invertebrates—Effects on nutrient turnover and soil structure—A review. Z. Pflanzenernahr. Bodenkd. [J. Plant Nutr. Soil Sci.].

[56] Bronick C.J., Lal R. (2005). Soil structure and management: A review. Geoderma.

[57] Katznelson H., Lochhead A.G., Timonin M.I. (1948). Soil microorganisms and the rhizosphere. Bot. Rev..

[58] Berg G., Smalla K. (2009). Plant species and soil type cooperatively shape the structure and function of microbial communities in the rhizosphere. FEMS Microbiol. Ecol..

[59] Jacoby R., Peukert M., Succurro A., Koprivova A., Kopriva S. (2017). The role of soil microorganisms in plant mineral nutrition-current knowledge and future directions. Front. Plant Sci..

[60] Fitzpatrick C.R., Copeland J., Wang P.W., Guttman D.S., Kotanen P.M., Johnson M.T.J. (2018). Assembly and ecological function of the root microbiome across angiosperm plant species. Proc. Natl. Acad. Sci. USA.

[61] Chirak E.L., Pershina E.V., Dolnik A.S., Kutovaya O.V., Vasilenko E.S., Kogut B.M., Merzlyakova Y.V., Andronov E.E. (2013). Taxonomic structure of microbial asso-ciation in differentsoils investigated by high-throughput sequencing of 16S-rRNAgene library. Sel’skokhozyaistvennaya Biol. [Agric. Biol.].

[62] Berg G., Rybakova D., Fischer D., Cernava T., Vergès M.C., Charles T., Chen X., Cocolin L., Eversole K., Corral G.H. (2020). Microbiome definition re-visited: Old concepts and new challenges. Microbiome.

[63] Van Bruggen A.H.C., Semenov A.M. (2000). In search of biological indicators for soil health and disease suppression. Appl. Soil Ecol..

[64] Evans K., Webster R., Barker A., Russel M., Stafford J., Griffin S. (2003). Mapping infestations of potato cyst nematodes and the potential for spatially varying application of nematicides. Precis. Agric..

[65] Goswami B.K., Chatterjee S., Singh N. (2017). Management strategies to rescue transplantable vegetables in and around yamuna river belt against heavy metals contamination and soil borne hidden enemies-a matter of great concern to human health. Plant Arch..

[66] Brodie B.B. (1976). Vertical distribution of three nematode species in relation to certain soil properties. J. Nematol..

[67] Noe J.P., Barker K.R. (1985). Relation of within-field spatial variation of plant-parasitic nematode population densities and edaphic factors. Phytopathology.

[68] Howland A.D., Schreiner R.P., Zasada I.A. (2014). Spatial distribution of plant-parasitic nematodes in semi-arid *Vitis vinifera* vineyards in Washington. J. Nematol..

[69] Kandel S.L., Smiley R.W., Garland-Campbell K., Elling A.A., Huggins D., Paulitz T.C. (2018). Spatial distribution of root lesion nematodes (*Pratylenchus* spp.) in a long-term no-till cropping system and their relationship with soil and landscape properties. Eur. J. Plant Pathol..

[70] Kawanobe M., Sugihara S., Miyamaru N., Yoshida K., Nonomura E., Oshiro H., Toyota K. (2020). Distribution of root-lesion and stunt nematodes, and their relationship with soil properties and nematode fauna in sugarcane fields in Okinawa, Japan. Agronomy.

[71] Mondal S., Sarkar P., Singh A., Khan M.R., Mukherjee A. (2019). Distribution and community structure of plant-parasitic nematodes and their relationship with some soil properties in betel vine-growing regions of West Bengal, India. Nematology.

[72] Krif G., Mokrini F., Aissami A.E., Laasli S.-E., Imren M., Özer G., Paulitz T., Lahlali R., Dababat A.A. (2020). Diversity and management strategies of plant parasitic nematodes in Moroccan organic farming and their relationship with soil physico-chemical properties. Agriculture.

[73] Avendaño F., Pierce F.J., Schabenberger O., Melakeberhan H. (2004). The spatial distribution of soybean cyst nematode in relation to soil texture and soil map unit. Agron. J..

[74] Hbirkou C., Welp G., Rehbein K., Hillnhütter C., Daub M., Oliver M.A., Pätzold S. (2011). The effect of soil heterogeneity on the spatial distribution of *Heterodera schachtii* within sugar beet fields. Appl. Soil Ecol..

[75] Holguin C.M., Gerard P., Mueller J.D., Khalilian A., Agudelo P. (2015). Spatial distribution of reniform nematode in cotton as influenced by soil texture and crop rotations. Phytopathology.

[76] Ortiz B.V., Perry C., Goovaerts P., Vellidis G., Sullivan D. (2010). Geostatistical modeling of the spatial variability and risk areas of southern root-knot nematodes in relation to soil properties. Geoderma.

[77] Yavuzaslanoglu E., Elekcioglu H.I., Nicol J.M., Yorgancilar O., Hodson D., Yildirim A.F., Yorgancilar A., Bolat N. (2012). Distribution, frequency and occurrence of cereal nematodes on the Central Anatolian Plateau in Turkey and their relationship with soil physicochemical properties. Nematology.

[78] Quist C.W., Gort G., Mooijman P., Brus D.J., van den Elsen S., Kostenko O., Vervoort M., Bakker J., van der Putten W.H., Helder J. (2019). Spatial distribution of soil nematodes relates to soil organic matter and life strategy. Soil Biol. Biochem..

[79] Archidona-Yuste A., Wiegand T., Castillo P., Navas-Cortes J.A. (2020). Spatial structure and soil properties shape local community structure of plant-parasitic nematodes in cultivated olive trees in southern Spain. Agric. Ecosyst. Environ..

[80] Briones M.J.I. (2018). The serendipitous value of soil fauna in ecosystem functioning: The unexplained explained. Front. Environ. Sci..

[81] Signorini M., Borruso L., Randall K.C., Dumbrell A.J., Pii Y., Mimmo T., Cesco S. (2021). Soil heterogeneity within a vineyard impacts the beta but not the alpha microbial agro-diversity. Appl. Soil Ecol..

[82] Curd E.E., Martiny J.B.H., Li H., Smith T.B. (2018). Bacterial diversity is positively correlated with soil heterogeneity. Ecosphere.

[83] Cheeke T.E., Schütte U.M., Hemmerich C.M., Cruzan M.B., Rosenstiel T.N., Bever J.D. (2015). Spatial soil heterogeneity has a greater effect on symbiotic arbuscular mycorrhizal fungal communities and plant growth than genetic modification with *Bacillus thuringiensis* toxin genes. Mol. Ecol..

[84] Mann C., Lynch D., Fillmore S., Mills A. (2019). Relationships between field management, soil health, and microbial community composition. Appl. Soil Ecol..

[85] Kandasamy S., Weerasuriya N., Subramanian G., Thorn R.G., Patterson G., Ali S., Lazarovits G. (2021). Disentangling the association of corn root mycobiome with plant productivity and the importance of soil physicochemical balance in shaping their relationship. Front. Sustain. Food Syst..

[86] Culman S.W., Young-Mathews A., Hollander A.D., Ferris H., Sánchez-Moreno S., O’Geen A., Jackson L. (2010). Biodiversity is associated with indicators of soil ecosystem functions over a landscape gradient of agricultural intensification. Landsc. Ecol..

[87] Steel H., Ferris H. (2016). Soil nematode assemblages indicate the potential for biological regulation of pest species. Acta Oecologica.

[88] Baker K., Cook R.J. (1974). Biological Control of Plant Pathogens.

[89] De Corato U. (2020). Disease-suppressive compost enhances natural soil suppressiveness against soil-borne plant pathogens: A critical review. Rhizosphere.

[90] Topalović O., Bredenbruch S., Schleker A.S.S., Heuer H. (2020). Microbes attaching to endoparasitic phytonematodes in soil trigger plant defense upon root penetration by the nematode. Front. Plant Sci..

[91] Weller D.M., Raaijmakers J.M., McSpadden Gardener B.B., Thomashow L.S. (2002). Microbial populations responsible for specific soil suppressiveness to plant pathogens. Ann. Rev. Phytopathol..

[92] Schlatter D., Kinkel L., Thomashow L., Weller D., Paulitz T. (2017). Disease suppressive soils: New insights from the soil microbiome. Phytopathology.

[93] Raaijmakers J.M., Mazzola M. (2016). Soil immune responses. Science.

[94] Postma J., Schilder M.T., Bloem J., van Leeuwen-Haagsma W.K. (2008). Soil suppressiveness and functional diversity of the soil microflora in organic farming systems. Soil Biol. Biochem..

[95] Da Silva J.C.P., de Medeiros F.H.V., Campos V.P. (2018). Building soil suppressiveness against plant-parasitic nematodes. Biocontrol Sci. Technol..

[96] Toyota K., Shirai S. (2018). Growing interest in microbiome research unraveling disease suppressive soils against plant pathogens. Microbes Environ..

[97] Mendes R., Kruijt M., De Bruijn I., Dekkers E., Van Der Voort M., Schneider J.H.M., Piceno Y.M., DeSantis T.Z., Andersen G.L., Bakker P.A.H.M. (2011). Deciphering the rhizosphere microbiome for disease-suppressive bacteria. Science.

[98] Adam M., Westphal A., Hallmann J., Heuer H. (2014). Specific microbial attachment to root knot nematodes in suppressive soil. Appl. Environ. Microbiol..

[99] Pyrowolakis A., Westphal A., Sikora R.A., Ole Becker J. (2002). Identification of root-knot nematode suppressive soils. Appl. Soil Ecol..

[100] McSorley R., Wang K., Church G. (2006). Effects of soil type and steam on nematode biological control potential of the rhizosphere community. Nematropica.

[101] Elhady A., Giné A., Topalović O., Jacquiod S., Sørensen S.J., Sorribas F.J., Heuer H. (2017). Microbiomes associated with infective stages of root-knot and lesion nematodes in soil. PLoS ONE.

[102] Hussain M., Hamid M.I., Tian J., Hu J., Zhang X., Chen J., Xiang M., Liu X. (2018). Bacterial community assemblages in the rhizosphere soil, root endosphere and cyst of soybean cyst nematode-suppressive soil challenged with nematodes. FEMS Microbiol. Ecol..

[103] Giné A., Carrasquilla M., Martínez-Alonso M., Gaju N., Sorribas F.J. (2016). Characterization of soil suppressiveness to root-knot nematodes in organic horticulture in plastic greenhouse. Front. Plant Sci..

[104] Siegel-Hertz K., Edel-Hermann V., Chapelle E., Terrat S., Raaijmakers J.M., Steinberg C. (2018). Comparative microbiome analysis of a Fusarium wilt suppressive soil and a Fusarium wilt conducive soil from the Châteaurenard region. Front. Microbiol..

[105] Lutz S., Thuerig B., Oberhaensli T., Mayerhofer J., Fuchs J.G., Widmer F., Freimoser F.M., Ahrens C.H. (2020). Harnessing the microbiomes of suppressive composts for plant protection: From metagenomes to beneficial microorganisms and reliable diagnostics. Front. Microbiol..

[106] Xiong W., Guo S., Jousset A., Zhao Q., Wu H., Li R., Kowalchuk G.A., Shen Q. (2017). Bio-fertilizer application induces soil suppressiveness against Fusarium wilt disease by reshaping the soil microbiome. Soil Biol. Biochem..

[107] Expósito R.G., de Bruijn I., Postma J., Raaijmakers J.M. (2017). Current insights into the role of Rhizosphere bacteria in disease suppressive soils. Front. Microbiol..

[108] Mazzola M. (2004). Assessment and management of soil microbial community structure for disease suppression. Ann. Rev. Phytopathol..

[109] Janvier C., Villeneuve F., Alabouvette C., Edel-Hermann V., Mateille T., Steinberg C. (2007). Soil health through soil disease suppression: Which strategy from descriptors to indicators?. Soil Biol. Biochem..

[110] Mazzola M., Freilich S. (2017). Prospects for biological soilborne disease control: Application of indigenous versus synthetic microbiomes. Phytopathology.

[111] Hoornweg D., Perinaz B.-T. (2012). What a Waste: A Global Review of Solid Waste Management.

[112] Soobhany N. (2018). Assessing the physicochemical properties and quality parameters during composting of different organic constituents of Municipal Solid Waste. J. Environ. Chem. Eng..

[113] Abad V., Avila R., Vicent T., Font X. (2019). Promoting circular economy in the surroundings of an organic fraction of municipal solid waste anaerobic digestion treatment plant: Biogas production impact and economic factors. Bioresour. Technol..

[114] Seruga P. (2021). The municipal solid waste management system with anaerobic digestion. Energies.

[115] Zaccheo P., Ricca G., Crippa L. (2002). Organic matter characterization of composts from different feedstocks. Compost Sci. Util..

[116] Brewer L.J., Sullivan D.M. (2003). Maturity and stability evaluation of composted yard trimmings. Compost Sci. Util..

[117] Kumar M., Ou Y.-L., Lin J.-G. (2010). Co-composting of green waste and food waste at low C/N ratio. Waste Manag..

[118] Azadi S., Karimi-Jashni A., Talebbeydokhti N., Khoshbakht R., Haghighi A.B. (2020). Industrial composting of commingled municipal solid waste: A case study of Shiraz City, Iran. J. Environ. Treat. Tech..

[119] Krause P., Oetjen-Dehne R., Dehne I., Dehnen D., Erchinger H. (2014). Compulsory Implementation of Separate Collection of Bio-Waste.

[120] Eades P., Kusch-Brandt S., Heaven S., Banks C.J. (2020). Estimating the generation of garden waste in England and the differences between rural and urban areas. Resources.

[121] Haigh M., Desai M., Cullis M., D’Aucourt M., Sansom B., Wilding G., Alun E., Garate S., Hatton L., Kilmartin M. (2019). Composted municipal green waste enhances tree success in opencast coal land reclamation in Wales. Air Soil Water Res..

[122] Boldrin A., Christensen T.H. (2010). Seasonal generation and composition of garden waste in Aarhus (Denmark). Waste Manag..

[123] Petrikovszki R., Zalai M., Tóthné Bogdányi F., Tóth F. (2020). The effect of organic mulching and irrigation on the weed species composition and the soil weed seed bank of tomato. Plants.

[124] Schüch A., Morscheck G., Lemke A., Nelles M. (2016). Bio-waste recycling in Germany—Further challenges. Procedia Environ. Sci..

[125] Agdag O.N. (2009). Comparison of old and new municipal solid waste management systems in Denizli, Turkey. J. Waste Manag..

[126] Hargreaves J.C., Adl M.S., Warman P.R. (2008). A review of the use of composted municipal solid waste in agriculture. Agric. Ecosyst. Environ..

[127] Communication from the Commission to the European Parliament, the Council, The European Economic and Social Committee and the Committee of the Regions (2020). A New Circular Economy Action Plan—For a Cleaner and More Competitive Europe.

[128] Eurostat. https://ec.europa.eu/eurostat/statistics-explained/index.php/Municipal_waste_statistics.

[129] Oliveira L.S., Oliveira D.S., Bezerra B.S., Pereira B.S., Battistelle R.A.G. (2017). Environmental analysis of organic waste treatment focusing on composting scenarios. J. Clean. Prod..

[130] Belyaeva O.N., Haynes R.J. (2009). Chemical, microbial and physical properties of manufactured soils produced by co-composting municipal green waste with coal fly ash. Bioresour. Technol..

[131] Belyaeva O.N., Haynes R.J. (2010). A comparison of the properties of manufactured soils produced from composting municipal green waste alone or with poultry manure or grease trap/septage waste. Biol. Fertil. Soils.

[132] Kabir M.J., Chowdhury A.A., Rasul M.G. (2015). Pyrolysis of Municipal Green Waste: A Modelling, Simulation and Experimental Analysis. Energies.

[133] Oliver G. (2009). Efficiency of energy recovery from municipal solid waste and the resultant effect on the greenhouse gas emission. Waste Manag. Res..

[134] Fazekas I., Szabó G., Szabó S., Paládi M., Szabó G., Buday T., Túri Z., Kerényi A. (2013). Biogas utilization and its environmental benefits in Hungary. Int. Rev. Appl. Sci. Eng..

[135] Slater R.A., Frederickson J. (2001). Composting municipal waste in the UK: Some lessons from Europe. Resour. Conserv. Recycl..

[136] Adams J.D.W., Zinnaro M., Frostick L.E. (2008). Composting of green waste: Observations from windrow trials and bench-scale experiments. Environ. Technol..

[137] Belyaeva O.N., Haynes R.J. (2012). Use of inorganic wastes as immobilizing agents for soluble P in green waste-based composts. Environ. Sci. Pollut. Res..

[138] Francou C., Lineres M., Derenne S., Le Villio-Poitrenaud M., Houot S. (2008). Influence of green waste, biowaste and paper-cardboard initial ratios on organic matter transformations during composting. Bioresour. Technol..

[139] Farrell M., Jones D.L. (2010). Food waste composting: Its use as a peat replacement. Waste Manag..

[140] Sim E.Y., Wu T.Y. (2010). The potential reuse of biodegradable municipal solid wastes (MSW) as feedstocks in vermicomposting. J. Sci. Food Agric..

[141] Lobl F., Stikova A., Stifter M., Vana J. (1977). Technological aspects of use of crushed solid town wastes and course of ripening of commercial composts. Rostl. Vyrob..

[142] Sayara T., Basheer-Salimia R., Hawamde F., Sánchez A. (2020). Recycling of organic wastes through composting: Process performance and compost application in agriculture. Agronomy.

[143] Abawi G.S., Widmer L. (2000). Impact of soil health management practices on soilborne pathogens, nematodes and root diseases of vegetable crops. Appl. Soil Ecol..

[144] Postma J., Montanari M., van den Boogert P.H.J.F. (2003). Microbial enrichment to enhance the disease suppressive activity of compost. Eur. J. Soil Biol..

[145] Hlava J. (2015). Soil fauna diversity relationship with NO_3_ content in grass filter strips within intensive aagriculture land. Pol. J. Ecol..

[146] Huang D.L., Zeng G.M., Feng C.L., Hu S., Lai C., Zhao M.H., Su F.F., Tang L., Liu H.L. (2010). Changes of microbial population structure related to lignin degradation during lignocellulosic waste composting. Bioresor. Technol..

[147] Das A., Baiswar P., Patel D.P., Munda G.C., Ghosh P.K., Ngachan S.V., Panwar A.S., Chandra S. (2010). Compost quality prepared from locally available plant biomass and their effect on rice productivity under organic production system. J. Sustain. Agric..

[148] Tognetti C., Mazzarino M.J., Laos F. (2008). Compost of municipal organic waste: Effects of different management practices on degradability and nutrient release capacity, *Soil Biol*. Biochem..

[149] Haynes R.J., Belyaeva O.N., Zhou Y.-F. (2015). Particle size fractionation as a method for characterizing the nutrient content of municipal green waste used for composting. Waste Manag..

[150] Sivapalan A., Morgan W.C., Franz P.R. (1993). Monitoring populations of soil-microorganisms during a conversion from a conventional to a organic-system of vegetable growing. Biol. Agric. Hortic..

[151] Allison S.D., LeBauer D.S., Ofrecio M.R., Reyes R., Ta A.M., Tran T.M. (2009). Low levels of nitrogen addition stimulate decomposition by boreal forest fungi. Soil Biol. Biochem..

[152] McLean M.A., Migge-Kleian S., Parkinson D. (2006). Earthworm invasions of ecosystems devoid of earthworms: Effects on soil microbes. Biol. Invasions.

[153] Breznak J.A., Brune A. (1994). Role of microorganisms in the digestion of lignocellulose by termites. Annu. Rev. Entomol..

[154] D’Hose T., Cougnon M., De Vliegher A., Vandecasteele B., Viaene N., Cornelis W., Van Bockstaele E., Reheul D. (2014). The positive relationship between soil quality and crop production: A case study on the effect of farm compost application. Appl. Soil Ecol..

[155] Nair A., Ngouajio M. (2012). Soil microbial biomass, functional microbial diversity, and nematode community structure as affected by cover crops and compost in an organic vegetable production system. Appl. Soil Ecol..

[156] Bernal M.P., Sommer S.G., Chadwick D., Qing C., Guoxue L., Michel F.C., Sparks D.L. (2017). Current approaches and future trends in compost quality criteria for agronomic, environmental, and human health benefits. Advances in Agronomy.

[157] Soobhany N. (2019). Insight into the recovery of nutrients from organic solid waste through biochemical conversion processes for fertilizer production: A review. J. Clean. Prod..

[158] Somerville P.D., Farrel C., May P.B., Livesley S.J. (2020). Biochar and compost equally improve urban soil physical and biological properties and tree growth, with no added benefit in combination. Sci. Total Environ..

[159] Bertola M., Ferrarini A., Visioli G. (2021). Improvement of soil microbial diversity through sustainable agricultural practices and its evaluation by -Omics approaches: A perspective for the environment, food quality and human safety. Microorganisms.

[160] Ros M., Klammer S., Knapp B., Aichberger K., Insam H. (2006). Long-term effects of compost amendment of soil on functional and structural diversity and microbial activity. Soil Use Manag..

[161] Przemieniecki S.W., Zapałowska A., Skwiercz A., Damszel M., Telesiński A., Sierota Z., Gorczyca A. (2021). An evaluation of selected chemical, biochemical, and biological parameters of soil enriched with vermicompost. Environ. Sci. Pollut. Res. Int..

[162] Pot S., De Tender C., Ommeslag S., Delcour I., Ceusters J., Gorrens E., Debode J., Vandecasteele B., Vancampenhout K. (2021). Understanding the shift in the microbiome of composts that are optimized for a better fit-for-purpose in growing media. Front. Microbiol..

[163] Borrero C., Trillas M.I., Ordovás J., Tello J.C., Avilés M. (2004). Predictive factors for the suppression of Fusarium wilt of tomato in plants growth media. Phytopathology.

[164] Haynes R.J., Zhou Y.-F. (2016). Comparison of the chemical, physical and microbial properties of composts produced by conventional composting or vermicomposting using the same feedstocks. Environ. Sci. Pollut. Res..

[165] Gomez-Brandon M., Lazcano C., Dominguez J. (2008). The evaluation of stability and maturity during the composting of cattle manure. Chemosphere.

[166] Hoitink H.A.J., Boehm M.J. (1999). Biocontrol within the context of soil microbial communities: A substrate-dependent phenomenon. Annu. Rev. Phytopathol..

[167] Sharon E., Bar-Eyal M., Chet I., Herrera-Estrella A., Kleifeld O., Spiegel Y. (2001). Biological control of the root-knot nematode *Meloidogyne javanica* by *Trichoderma harzianum*. Phytopathology.

[168] Kluepfel D.A., Nyczepir A.P., Lawrence J.E., Wechter W.P., Leverentz B. (2002). Biological control of the phytoparasitic nematode *Mesocriconema xenoplax* on peach trees. J. Nematol..

[169] Mekete T., Hallmann J., Kiewnick S., Sikora R. (2009). Endophytic bacteria from Ethiopian coffee plants and their potential to antagonize *Meloidogyne incognita*. Nematology.

[170] Kouki S., Saidi N.A., Rajeb B., Brahmi M., Bellila A., Fumio M., Hefiène A., Jedidi N., Downer J., Ouzari H. (2012). Control of fusarium wilt of tomato caused by *Fusarium oxysporum* f. sp. radicis-lycopersici using mixture of vegetable and *Posidonia oceanica* compost. Appl. Environ. Soil Sci..

[171] El Khaldi R., Daami-Remadi M., Hamada W., Somai L., Cherif M. (2015). The potential of *Serratia marcescens*: An indigenous strain isolated from date palm compost as biocontrol agent of *Rhizoctonia solani* on potato. J. Plant Pathol. Microbiol..

[172] Malandraki I., Tjamos S.E., Pantelides I.S., Paplomatas E.J. (2008). Thermal inactivation of compost suppressiveness implicates possible biological factors in disease management. Biol. Control.

[173] Daami-Remadi M., Dkhili I., Jabnoun-Khiareddine H., El Mahjoub M. (2012). Biological control of potato leak with antagonistic fungi isolated from compost teas and solarized and non-solarized soils. Pest Technol..

[174] Daragó Á., Szabó M., Hrács K., Takács A., Nagy P. (2013). In vitro investigations on the biological control of *Xiphinema index* with *Trichoderma* species. Helminthologia.

[175] Chen S., Dickson D.W., Chen Z.X., Chen S.Y., Dickson D.W. (2004). Biological control of nematodes by fungal antagonists. Nematology Advances and Perspectives, Vol. 2, Nematode Management and Utilization.

[176] Kumar N., Singh R.K., Singh K.P. (2011). Occurrence and colonization of nematophagous fungi in different substrates, agricultural soils and root galls. Arch. Phytopathol. Plant Prot..

[177] Kiontke K., Fitch H.A.D. (2013). Nematodes. Curr. Biol..

[178] Wang X., Li G.H., Zou C.G., Ji X., Liu T., Zhao P.J., Liang L.M., Xu J.P., An Z.Q., Zheng X. (2014). Bacteria can mobilize nematode-trapping fungi to kill nematodes. Nat. Commun..

[179] Liang L.M., Zou C.G., Xu J., Zhang K.Q. (2019). Signal pathways involved in microbe-nematode interactions provide new insights into the biocontrol of plant-parasitic nematodes. Philos. Trans. R. Soc. Lond. Ser. B Biol. Sci..

[180] Bordallo J.J., Lopez-Llorca L.V., Jansson H.-B., Salinas J., Persmark L., Asensio L. (2002). Colonization of plant roots by egg-parasitic and nematode-trapping fungi. New Phytol..

[181] Szabó M., Csepregi K., Gálber M., Virányi F., Fekete C. (2012). Control plant-parasitic nematodes with *Trichoderma* species and nematode-trapping fungi: The role of chi18–5 and chi18–12 genes in nematode egg-parasitism. Biol. Control.

[182] Cumagun C.J.R., Moosavi M.R., Askary T.H., Martinelli P.R.P. (2015). Significance of biocontrol agents of phytonematodes. Biocontrol Agents of Phytonematodes.

[183] Maheshwari D.K., Shukla S., Aeron A., Kumar T., Jha C.K., Patel D., Saraf M., Wahla V., Maheshwari D.K. (2013). Rhizobacteria for management of nematode disease in plants. Bacteria in Agrobiology: Disease Management.

[184] Khan Z., Kim S.G., Jeon Y.H., Khan H.U., Son S.H., Kim Y.H. (2008). A plant growth promoting rhizobacterium, *Paenibacillus polymyxa* strain GBR-1, suppresses root-knot nematode. Bioresour. Technol..

[185] Abd-Elgawad M.M.M., Askary T.H. (2018). Fungal and bacterial nematicides in integrated nematode management strategies. Egypt. J. Biol. Pest Control.

[186] Ansari R.A., Mahmood I., Rizvi R., Sumbul A., Kumar V., Kumar M., Sharma S., Prasad R. (2017). Siderophores: Augmentation of soil health and crop productivity. Probiotics in Agroecosystem.

[187] Hallmann J., Davies K.G., Sikora R., Hallmann J., Davies K.G., Sikora R. (2009). Biological control using microbial pathogens, endophytes and antagonists. Root-Knot Nematodes.

[188] Ansari R.A., Rizvi R., Sumbul A., Mahmood I., Kumar V., Kumar M., Sharma S., Prasad R. (2017). Current vogue in sustainable crop production. Probiotics and Plant Health.

[189] Wislocki P.G., Grosso L.S., Dybas R.A., Campbell W.C. (1989). Environmental aspects of abamectin use in crop protection. Ivermectin and Abamectin.

[190] Aktuganov G., Melentjev A., Galimzianova N., Khalikova E., Korpela T., Susi P. (2008). Wide-range antifungal antagonism of *Paenibacillus ehimensis* IB-X-b and its dependence on chitinase and β-1,3-glucanase production. Can. J. Microbiol..

[191] Yang J., Kharbanda P.D., Mirza M. (2004). Evaluation of *Paenibacillus polymyxa* PKB1 for biocontrol of pythium disease of cucumber in a hydroponic system. Acta Hortic..

[192] Tian B., Yang J., Zhang K.Q. (2007). Bacteria used in the biological control of plant-parasitic nematodes: Populations, mechanisms of action, and future prospects. FEMS Microbiol. Ecol..

[193] Zhai Y., Shao Z., Cai M., Zheng L., Li G., Huang D., Cheng W., Thomashow L.S., Weller D.M., Yu Z. (2018). Multiple modes of nematode control by volatiles of *Pseudomonas putida* 1A00316 from Antarctic soil against *Meloidogyne incognita*. Front. Microbiol..

[194] Chang E.H., Chung R.S., Tsai Y.H. (2007). Effect of different application rates of organic fertilizer on soil enzyme activity and microbial population: Original article. Soil Sci. Plant Nutr..

[195] Usman A., Siddiqui M.A. (2013). Integrated approaches of phytonematodes management by organic soil amendments and ploughing. Pak. J. Nematol..

[196] Akhtar M., Malik A. (2000). Roles of organic soil amendments and soil organisms in the biological control of plant-parasitic nematodes: A review. Bioresour. Technol..

[197] Dutta T.K., Khan M.R., Phani V. (2019). Plant-parasitic nematode management via biofumigation using brassica and non-brassica plants: Current status and future prospects. Curr. Plant Biol..

[198] Oka Y. (2010). Mechanisms of nematode suppression by organic soil amendments—A review. Appl. Soil Ecol..

[199] Hale L., Curtis D., Azeem M., Montgomery J., Crowley D.E., McGiffen M.E. (2021). Influence of compost and biochar on soil biological properties under turfgrass supplied deficit irrigation. Appl. Soil Ecol..

[200] Azeem M., Hale L., Montgomery J., Crowley D., McGiffen M.E. (2020). Biochar and compost effects on soil microbial communities and nitrogen induced respiration in turfgrass soils. PLoS ONE.

[201] Zhao C., Li Y., Li Y., Zhang C., Miao Y., Liu M., Zhuang W., Shao Y., Zhang W., Fu S. (2021). Considerable impacts of litter inputs on soil nematode community composition in a young *Acacia crassicapa* plantation. Soil Ecol. Lett..

[202] Kurzemann F.R., Plieger U., Probst M., Spiegel H., Sandén T., Ros M., Insam H. (2020). Long-term fertilization affects soil microbiota, improves yield and benefits soil. Agronomy.

[203] Hong S.H., Anees M., Kim K.Y. (2013). Biocontrol of *Meloidogyne incognita* inciting disease in tomato by using a mixed compost inoculated with *Paenibacillus ehimensis* RS820. Biocontrol Sci Technol..

[204] Achmon Y., Claypool J.T., Fernández-Bayo J.D., Hernandez K., McCurry D.G., Harrold D.R., Su J., Simmons B.A., Singer S.W., Dahlquist-Willard R.M. (2020). Structural changes in bacterial and fungal soil microbiome components during biosolarization as related to volatile fatty acid accumulation. Appl. Soil Ecol..

[205] Liang B., Ma C., Fan L., Wang Y., Yuan Y. (2021). Compost amendment alters soil fungal community structure of a replanted apple orchard. Arch. Agron. Soil Sci..

[206] Ntalli N., Adamski Z., Doula M., Monokrousos N. (2020). Nematicidal amendments and soil remediation. Plants.

[207] Ikwunagu E.A., Ononuju C.C., Orikara C.C. (2019). Nematicidal effects of dfferent biochar sources on root-knot nematode (*Meloidogyne* spp.) egg hatchability and control on mungbean (*Vigna radiata* (L.) Wilczek). Int. J. Entomol. Nematol. Res..

[208] Avato P., D’Addabbo T., Leonetti P., Argentieri M.P. (2013). Nematicidal potential of Brassicaceae. Phytochem. Rev..

[209] Kokalis-Burelle N., Rosskopf E.N., Iriarte F. (2009). Evaluation of spk, a novel combination of organic compounds for root-knot nematode control in tomato. J. Nematol..

[210] D’Addabbo T., Migunova V.D., Renčo M., Sasanelli N. (2020). Suppressiveness of soil amendments with pelleted plant materials on the root-knot nematode *Meloidogyne incognita*. Helminthologia.

[211] Mehta C.M., Palni U., Franke-Whittle I.H., Sharma A.K. (2014). Compost: Its role, mechanism and impact on reducing soil-borne plant diseases. Waste Manag..

[212] Du Jardin P. (2015). Plant biostimulants: Definition, concept, main categories and regulation. Sci. Hortic..

[213] Alori E.T., Babalola O.O. (2018). Microbial inoculants for improving crop quality and human health in Africa. Front. Microbiol..

[214] Pandey V., Chandra K., Singh H.B., Sarma B.K., Keswani C. (2016). Agriculturally important microorganisms as biofertilizers: Commercialization and regulatory requirements in Asia. Agriculturally Important Microorganisms: Commercialization and Regulatory Requirements in Asia.

[215] Kokalis-Burelle N., Vavrina C.S., Reddy M.S., Kloepper J.W. (2003). Amendment of muskmelon transplant media with plant growth-promoting rhizobacteria: Effects on seedling quality, disease, and nematode resistance. Hortechnology.

[216] Tall S., Meyling N.V. (2018). Probiotics for plants? Growth promotion by the entomopathogenic fungus *Beauveria bassiana* depends on nutrient availability. Microb. Ecol..

[217] Neher D.A., Weicht T.R., Bates S.T., Leff J.W., Fierer N. (2013). Changes in bacterial and fungal communities across compost recipes, preparation methods, and composting times. PLoS ONE.

[218] Sant D., Casanova E., Segarra G., Avilés M., Reis M., Trillas M.I. (2010). Effect of *Trichoderma asperellum* strain T34 on Fusarium wilt and water usage in carnation grown on compost-based growth medium. Biol. Control.

[219] Trillas M.I., Casanova E., Cotxarrera L., Ordovás J., Borrero C., Avilés M. (2006). Composts from agricultural waste and the *Trichoderma asperellum* strain T-34 suppress *Rhizoctonia solani* in cucumber seedlings. Biol. Control.

[220] Leggett M., Leland J., Kellar K., Epp B. (2011). Formulation of microbial control agents—An industrial perspective. Can. J. Plant Pathol..

[221] Hussain A., Rizwan-ul-Haq M., Al-Ayedh H., Al Jabr A.M. (2014). Mycoinsecticides: Potential and future perspective. Recent Pat. Food Nutr. Agric..

[222] Jaber L.R., Ownley B.H. (2018). Can we use entomopathogenic fungi as endophytes for dual biological control of insect pests and plant pathogens?. Biol. Control.

[223] Mascarin G.M., Lopes R.B., Delalibera Í., Fernandes É.K.K., Luz C., Faria M. (2019). Current status and perspectives of fungal entomopathogens used for microbial control of arthropod pests in Brazil. J. Invertebr. Pathol..

[224] Kergunteuil A., Bakhtiari M., Formenti L., Xiao Z., Defossez E., Rasmann S. (2016). Biological control beneath the feet: A review of crop protection against insect root herbivores. Insects.

[225] Abd-Elgawad M.M.M., Askary T.H. (2020). Factors affecting success of biological agents used in controlling the plant-parasitic nematodes. Egypt. J. Biol. Pest Control.

[226] Joos L., Herren G.L., Couvreur M., Binnemans I., Oni F.E., Höfte M., Debode J., Bert W., Steel H. (2020). Compost is a carrier medium for *Trichoderma harzianum*. BioControl.

[227] Herren G.L., Binnemans I., Joos L., Viaene N., Ehlers R.-U., Vandecasteele B., Bert W., Steel H. (2018). Compost as a carrier medium for entomopathogenic nematodes—The influence of compost maturity on their virulence and survival. Biol. Control.

[228] Ding S.W. (2000). RNA silencing. Curr. Opin. Biotechnol..

[229] Fire A., Xu S., Montgomery M.K., Kostas S.A., Driver S.E., Mello C.C. (1998). Potent and specific genetic interference by double-stranded RNA in *Caenorhabditis elegans*. Nature.

[230] Baulcombe D. (1999). Viruses and gene silencing in plants. Arch. Virol. Suppl..

[231] Leonetti P., Stuttmann J., Pantaleo V. (2021). Regulation of plant antiviral defense genes via host RNA-silencing mechanisms. Virol. J..

[232] Ahlquist P. (2002). RNA-dependent RNA polymerases, viruses, and RNA silencing. Science.

[233] Himber C., Dunoyer P., Moissiard G., Ritzenthaler C., Voinnet O. (2003). Transitivity-dependent and -independent cell to-cell movement of RNA silencing. EMBO J..

[234] Carbonell A., Carrington J.C. (2015). Antiviral roles of plant ARGONAUTES. Curr. Opin. Plant Biol..

[235] Pantaleo V., Szittya G., Burgyan J. (2007). Molecular bases of viral RNA targeting by viral small interfering RNA-programmed RISC. J. Virol..

[236] Cao M., Du P., Wang X., Yu Y.Q., Qiu Y.H., Li W., Gal-On A., Zhou C., Li Y., Ding S.W. (2014). Virus infection triggers widespread silencing of host genes by a distinct class of endogenous siRNAs in *Arabidopsis*. Proc. Natl. Acad. Sci. USA.

[237] Leonetti P., Ghasemzadeh A., Consiglio A., Gursinsky T., Behrens S., Pantaleo V. (2021). Endogenous activated small interfering RNAs in virus-infected Brassicaceae crops show a common host gene-silencing pattern affecting photosynthesis and stress response. New Phytol..

[238] Raja P., Sanville B.C., Buchmann R.C., Bisaro D.M. (2008). Viral genome methylation as an epigenetic defense against geminiviruses. J. Virol..

[239] Wang M., Weiberg A., Lin F.-M., Thomma B.P.H.J., Huang H.-D., Jin H. (2016). Bidirectional cross-kingdom RNAi and fungal uptake of external RNAs confer plant protection. Nat. Plants.

[240] Li J., Todd T.C., Lee J., Trick H.N. (2011). Biotechnological application of functional genomics towards plant-parasitic nematode control. Plant Biotechnol. J..

[241] Gheysen G., Vanholme B. (2007). RNAi from plants to nematodes. Trends Biotechnol..

[242] Rosso M.N., Jones J.T., Abad P. (2009). RNAi and functional genomics in plant parasitic nematodes. Annu. Rev. Phytopathol..

[243] Basso M.F., Lourenço-Tessutti I.T., Mendes R.A.G., Pinto C.E.M., Bournaud C., Gillet F.X., Togawa R.C., de Macedo L.L.P., de Almeida Engler J., Grossi-de-Sa M.F. (2020). MiDaf16-like and MiSkn1-like gene families are reliable targets to develop biotechnological tools for the control and management of *Meloidogyne incognita*. Sci. Rep..

[244] Blyuss K.B., Fatehi F., Tsygankova V.A., Biliavska L.O., Iutynska G.O., Yemets A.I., Blume Y.B. (2019). RNAi-based biocontrol of wheat nematodes using natural poly-component biostimulants. Front. Plant Sci..

[245] Tsygankova V.A., Andrusevich Y.V., Galkin A.P., Biliavska L.O., Galagan T.O., Yemets A.I., Iutynska G.A., Blume B.Y. (2019). RNAi-mediated resistance against plant parasitic nematodes of wheat plants obtained in vitro using bioregulators of microbiological origin. Curr. Chem. Biol..

[246] Gualtieri C., Leonetti P., Macovei A. (2020). Plant miRNA cross-kingdom transfer targeting parasitic and mutualistic organisms as a tool to advance modern agriculture. Front. Plant Sci..

[247] Cai Q., He B., Kogel K.H., Jin H. (2018). Cross-kingdom RNA trafficking and environmental RNAi-nature’s blueprint for modern crop protection strategies. Curr. Opin. Microbiol..

[248] Renčo M., Sasanelli N., D’Addabbo T., Papajová I. (2010). Soil nematode community changes associated with compost amendments. Nematology.

[249] Renčo M., Sasanelli N., Šalamún P. (2009). The effect of two compost soil amendments, based on municipal green and penicillin production wastes, on plant parasitic nematodes. Helminthologia.

[250] Akhtar M., Mahmood I. (1994). Potentiality of phytochemicals in nematode control: A review. Bioresour. Technol..

[251] Dama L.B. (2002). Effect of naturally occurring napthoquinones on root-knot nematode *Meioidogyne javanica*. Indian Phytopath..

[252] Dama L.B. (2019). In vitro nematicidal activity of Juglone against *Meloidogyne incognita* race 2 infesting pomegranate. J. Life Sci. Biomed..

[253] Jakusovszky R., Petrikovszki R., Kiss L.V., Bogdányi F.T., Tóth F., Nagy P.I. (2019). Ecotoxicological studies with aqueous extracts of walnut leaf litter on plant parasitic nematodes and on other test organisms. [Dióavar-kivonatok ökotoxikológiai vizsgálata növénykártevő fonálférgeken és más tesztszervezeteken]. Növényvédelem.

[254] Steel H., Moens T., Vandecasteele B., Hendrickx F., De Neve S., Neher D.A., Bert W. (2018). Factors influencing the nematode community during composting and nematode-based criteria for compost maturity. Ecol. Indic..

[255] Thoden T.C., Korthals G.W., Termorshuizen A.J. (2011). Organic amendments and their influences on plant-parasitic and free-living nematodes: A promising method for nematode management?. Nematology.

[256] Battini F., Grønlund M., Agnolucci M., Giovannetti M., Jakobsen I. (2017). Facilitation of phosphorus uptake in maize plants by mycorrhizosphere bacteria. Sci. Rep..

[257] Wang X.-X., Hoffland E., Feng G., Kuyper T.W. (2020). Arbuscular mycorrhizal symbiosis increases phosphorus uptake and productivity of mixtures of maize varieties compared to monocultures. J. Appl. Ecol..

[258] Johnson N.C., Graham J.H. (2013). The continuum concept remains a useful framework for studying mycorrhizal functioning. Plant Soil.

[259] Schouteden N., De Waele D., Panis B., Vos C.M. (2015). Arbuscular mycorrhizal fungi for the biocontrol of plant-parasitic nematodes: A review of the mechanisms involved. Front. Microbiol..

[260] Poveda K., Steffan-Dewenter I., Scheu S., Tscharntke T., Ohgushi T., Craig T., Price P. (2007). Plant-mediated interactions between below- and aboveground processes: Decomposition, herbivory, parasitism, and pollination. Ecological Communities: Plant Mediation in Indirect Interaction Webs.

[261] Cahill J.F., Elle E., Smith G.R., Shore B.H. (2008). Disruption of a belowground mutualism alters interactions between plants and their floral visitors. Ecology.

[262] Vos C., Schouteden N., van Tuinen D., Chatagnier O., Elsen A., De Waele D., Panis B., Gianinazzi-Pearson V. (2013). Mycorrhiza-induced resistance against the root–knot nematode *Meloidogyne incognita* involves priming of defense gene responses in tomato. Soil Biol. Biochem..

[263] Ferreira B.S., Santana M.V., Macedo R.S., Silva J.O., Carneiro M.A.C., Rocha M.R. (2018). Co-occurrence patterns between plant-parasitic nematodes and arbuscular mycorrhizal fungi are driven by environmental factors. Agric. Ecosyst. Environ..

[264] Benedetti T., Antoniolli Z.I., Sordi E., Carvalho I.R., Bortoluzzi E.C. (2021). Use of the *Glomus etunicatum* as biocontrol agent of the soybean cyst nematode. Res. Soc. Dev..

[265] Amerany F.E., Rhazi M., Wahbi S., Taourirte M., Meddich A. (2020). The effect of chitosan, arbuscular mycorrhizal fungi, and compost applied individually or in combination on growth, nutrient uptake, and stem anatomy of tomato. Sci. Hortic..

[266] Cavagnaro T.R. (2014). Impacts of compost application on the formation and functioning of arbuscular mycorrhizas. Soil Biol. Biochem..

[267] Jan B., Ali A., Wahid F., Shah S., Khan A., Khan F. (2014). Effect of arbuscular mycorrhiza fungal inoculation with compost on yield and phosphorous uptake of berseem in alkaline calcareous soil. Am. J. Plant Sci..

[268] Galal N.M., Morsyand E.M., Massoud O.N. (2012). Evaluation of some microbial biocontrol agents and compost against root-knot nematode (*Meloidogyne javanica*). N. Egypt. J. Microbiol..

[269] Rizvi R., Mahmood I., Ansari S. (2018). Interaction between plant symbionts, bio-organic waste and antagonistic fungi in the management of *Meloidogyne incognita* infecting chickpea. J. Saudi Soc. Agric. Sci..

[270] Wurst S. (2010). Effects of earthworms on above- and belowground herbivores. Appl. Soil Ecol..

[271] Xiao Z., Liu M., Jiang L., Chen X., Griffiths B.S., Li H., Hu F. (2016). Vermicompost increases defense against root-knot nematode (*Meloidogyne incognita*) in tomato plants. Appl. Soil Ecol..

[272] Gudeta K., Julka J.M., Kumar A., Bhagat A., Kumari A. (2021). Vermiwash: An agent of disease and pest control in soil, a review. Heliyon.

[273] Tao J., Chen X., Liu M., Hu F., Griffiths B., Li H. (2009). Earthworms change the abundance and community structure of nematodes and protozoa in a maize residue amended rice–wheat rotation agro-ecosystem. Soil Biol. Biochem..

[274] Demetrio W.C., Dionísió J.A., Maceda A. (2019). Negative effects of earthworms on soil nematodes are dependent on earthworm density, ecological category and experimental conditions. Pedobiologia.

[275] Marull J., Pinochet J., Rodrıguez-Kabana R. (1997). Agricultural and municipal compost residues for control of root-knot nematodes in tomato and pepper. Compost Sci. Util..

[276] Nelson E.B., Hoitink H.A.J. (1983). The role of microorganisms in the suppression of Rhizoctonia solani in composted hardwood bark container media. Phytopathology.

[277] Kuter G.A., Nelson E.B., Hoitink H.A.J., Madden L.V. (1983). Fungal populations in container media amended with composted hardwood bark suppressive and conducive to *Rhizoctonia* damping-off. Phytopathology.

[278] Dionne A., Tweddell R.J., Antoun H., Avis T.J. (2012). Effect of non-aerated compost teas on damping-off pathogens of tomato. Can. J. Plant Pathol..

[279] Alfano G., Lustrato G., Lima G., Vitullo D., Ranalli G. (2011). Characterization of composted olive mill wastes to predict potential plant disease suppressiveness. Biol. Control.

[280] Chen J., Abawi G.S., Zuckerman B.M. (2000). Efficacy of *Bacillus thuringiensis*, *Paecilomyces marquandii*, and *Streptomyces costaricanus* with and without organic amendments against *Meloidogyne hapla* infecting lettuce. J. Nematol..

[281] McSorley R., Gallaher R.N. (1995). Effect of yard waste compost on plant–parasitic nematode densities in vegetable crops. J. Nematol..

[282] McSorley R. (2011). Overview of organic amendments for management of plant-parasitic nematodes, with case studies from Florida. J. Nematol..

[283] Moosavi M.R., Zare R., Mérillon J.M., Ramawat K.G. (2020). Fungi as biological control agents of plant-parasitic nematodes. Plant Defence: Biological Control.

[284] Saxena G., Giri B., Prasad R., Varma A. (2018). Biological control of root-knot and cyst nematodes using nematophagous fungi. Root Biology.

[285] Westphal A., Becker J.O. (2001). Components of soil suppressiveness against *Heterodera schachtii*. Soil Biol. Biochem..

[286] Calvet C., Pinochet J., Hernández-Dorrego A. (2001). Field microplot performance of the peach almond hybrid GF-677 after inoculation with arbuscular mycorrhizal fungi in a replant soil infested with root-knot nematodes. Mycorrhizae.

[287] Zakaria H.M., Kassab A.S., Shamseldean M.M., Oraby M.M., El-Mourshedy M.M.F. (2013). Controlling the root-knot nematode, Meloidogyne incognita in cucumber plants using some soil bioagents and some amendments under simulated field conditions. Ann. Agric. Sci..

[288] Devi G., Bora L.C. (2018). Effect of some biocontrol agents against root-knot nematode (*Meloidogyne incognita* race2). Int. J. Environ. Agric. Biotechnol..

[289] Devi G. (2018). Nematophagous fungi: *Metarhizium anisopliae*. J. Environ. Agric. Biotechnol..

[290] Leoni C., Piancone E., Sasanelli N., Bruno G.L., Manzari C., Pesole G., Ceci L.R., Volpicella M. (2020). Plant health and rhizosphere microbiome: Effects of the bionematicide *Aphanocladium album* in tomato plants infested by *Meloidogyne javanica*. Microorganisms.

[291] Kumar K.K., Dara S.K. (2021). Fungal and bacterial endophytes as microbial control agents for plant-parasitic nematodes. Int. J. Environ. Res. Public Health.

[292] Spiegel Y., Chet I. (1998). Evaluation of *Trichoderma* spp. as a biocontrol agent against soilborne fungi and plant-parasitic nematodes in Israel. Integr. Pest Manag. Rev..

[293] Spiegel Y., Sharon E., Chet I. (2005). Mechanisms and improved biocontrol of the root-knot nematodes by *Trichoderma* spp.. Acta Hortic..

[294] Sahebani N., Hadavi N. (2008). Biological control of the root-knot nematode *Meloidogyne javanica* by *Trichoderma harzianum*. Soil Biol. Biochem..

[295] Khan M.R., Ahmad I., Ahamad F. (2018). Effect of pure culture and culture filtrates of *Trichoderma* species on root-knot nematode, *Meloidogyne incognita* infesting tomato. Indian Phytopathol..

[296] Leonetti P., Zonno M.C., Molinari S., Altomare C. (2017). Induction of SA-signaling pathway and ethylene biosynthesis in *Trichoderma harzianum*-treated tomato plants after infection of the root-knot nematode *Meloidogyne incognita*. Plant Cell Rep..

[297] Khan A., Williams K.L., Nevalainen H.K.M. (2006). Control of plant-parasitic nematodes by *Paecilomyces lilacinus* and *Monacrosporium lysipagum* in pot trials. Biocontrol.

[298] Kiewnick S., Sikora R.A. (2006). Biological control of the root-knot nematode *Meloidogyne incognita* by *Paecilomyces lilacinus* strain 251. Biol. Control.

[299] Kiewnick S., Sikora R.A. (2006). Evaluation of *Paecilomyces lilacinus* strain 251 for the biological control of the northern root-knot nematode *Meloidogyne hapla* Chitwood. Nematology.

[300] Youssef M.M.A., Eissa M.F.M. (2014). Biofertilizers and their role in management of plant parasitic nematodes. A review. E3 J. Biotechnol. Pharm. Res..

[301] Topalović O., Elhady A., Hallmann J., Richert-Pöggeler K.R., Heuer H. (2019). Bacteria isolated from the cuticle of plant-parasitic nematodes attached to and antagonized the root-knot nematode *Meloidogyne hapla*. Sci. Rep..

[302] Abd-Elgawad M.M.M., Ansari R., Rizvi R., Mahmood I. (2020). Plant-parasitic nematodes and their biocontrol agents: Current status and future vistas. Management of Phytonematodes: Recent Advances and Future Challenges.

[303] Mhatre P.H., Karthik C., Kadirvelu K., Divya K.L., Venkatasalam E.P., Srinivasan S., Ramkumar G., Saranya C., Shanmuganathan R. (2018). Plant Growth Promoting Rhizobacteria (PGPR): A potential alternative tool for Nematodes bio-control. Biocatal. Agric. Biotechnol..

[304] Abuzar S., Haseeb A. (2010). Plant growth and plant parasitic nematodes in response to soil amendments with Plant Growth Promoting Rhizobacteria and inorganic fertilizer in pigeon pea, *Cajanus cajan* L.. World Appl. Sci. J..

[305] Khanna K., Jamwal V.L., Kohli S.K., Gandhi S.G., Ohri P., Bhardwaj R., Wijaya L., Alyemeni M.N., Ahmad P. (2019). Role of plant growth promoting Bacteria (PGPRs) as biocontrol agents of *Meloidogyne incognita* through improved plant defense of *Lycopersicon esculentum*. Plant Soil.

[306] Khanna K., Sharma A., Ohri P., Bhardwaj R., Abd Allah E.F., Hashem A., Ahmad P. (2019). Impact of plant growth promoting Rhizobacteria in the orchestration of *Lycopersicon esculentum* Mill. resistance to plant parasitic nematodes: A metabolomic approach to evaluate defense responses under field conditions. Biomolecules.

[307] Wilson M.J., Jackson T.A. (2013). Progress in the commercialization of bionematicides. BioControl.

[308] Chandrashekara C., Kumar R., Bhatt J.C., Chandrashekara K., Singh V.K., Singh Y., Singh A. (2012). Suppressive soils in plant disease management. Eco-Friendly Innovative Approaches in Plant Disease Management.

[309] Renčo M., Kováčik P. (2015). Assessment of the nematicidal potential of vermicompost, vermicompost tea, and urea application on the potato-cyst nematodes *Globodera rostochiensis* and *Globodera pallida*. J. Plant Protect. Res..

[310] Litterick A.M., Harrier L., Wallace P., Watson C.A., Wood M. (2004). The role of uncomposted materials, composts, manures, and compost extracts in reducing pest and disease incidence and severity in sustainable temperate agricultural and horticultural crop production—A review. Crit. Rev. Plant Sci..

[311] St. Martin C.C.G., Ramsubhag A., Ganesan S., Vadivel K., Jayaraman J. (2015). Potential of compost for suppressing plant diseases. Sustainable Crop Disease Management Using Natural Products.

[312] Bonanomi G., Lorito M., Vinae F., Woo S. (2018). Organic amendments, beneficial microbes, and soil microbiota: Toward a unified framework for disease suppression. Annu. Rev. Phytopathol..

[313] De Corato U. (2020). Soil microbiota manipulation and its role in suppressing soil-borne plant pathogens in organic farming systems under the light of microbiome-assisted strategies. Chem. Biol. Technol. Agric..

[314] Ghini R., Boechat Morandi M.A. (2006). Biotic and abiotic factors associated with soil suppressiveness to *Rhizoctonia solani*. Sci. Agric..

[315] Zhou D., Feng H., Schuelke T., De Santiago A., Zhang Q., Zhang J., Luo C., Wei L. (2019). Rhizosphere microbiomes from root knot nematode non-infested plants suppress nematode infection. Microb. Ecol..

[316] Berg G., Köberl M., Rybakova D., Müller H., Grosch R., Smalla K. (2017). Plant microbial diversity is suggested as the key to future biocontrol and health trends. FEMS Microbiol. Ecol..

